# Infection, delirium, and risk of dementia in patients with and without white matter disease on previous brain imaging: a population-based study

**DOI:** 10.1016/S2666-7568(23)00266-0

**Published:** 2024-02-01

**Authors:** Sarah T Pendlebury, Ramon Luengo-Fernandez, Anna Seeley, Matthew B Downer, Aubretia McColl, Peter M Rothwell, for the Oxford Vascular Study

**Affiliations:** Wolfson Centre for Prevention of Stroke and Dementia, Wolfson Building, Nuffield Department of Clinical Neurosciences, John Radcliffe Hospital; University of Oxford, Oxford, UK; NIHR Oxford Biomedical Research Centre, Oxford University Hospitals NHS Foundation Trust, Oxford, UK; Departments of Acute General Internal Medicine and Geratology, Oxford University Hospitals NHS Foundation Trust, Oxford, UK; Wolfson Centre for Prevention of Stroke and Dementia, Wolfson Building, Nuffield Department of Clinical Neurosciences, John Radcliffe Hospital; Wolfson Centre for Prevention of Stroke and Dementia, Wolfson Building, Nuffield Department of Clinical Neurosciences, John Radcliffe Hospital; Nuffield Department of Primary Care Health Sciences; Wolfson Centre for Prevention of Stroke and Dementia, Wolfson Building, Nuffield Department of Clinical Neurosciences, John Radcliffe Hospital; Wolfson Centre for Prevention of Stroke and Dementia, Wolfson Building, Nuffield Department of Clinical Neurosciences, John Radcliffe Hospital; Wolfson Centre for Prevention of Stroke and Dementia, Wolfson Building, Nuffield Department of Clinical Neurosciences, John Radcliffe Hospital; University of Oxford, Oxford, UK; NIHR Oxford Biomedical Research Centre, Oxford University Hospitals NHS Foundation Trust, Oxford, UK

## Abstract

**Background:**

The increased risk of dementia after delirium and infection might be influenced by cerebral white matter disease (WMD). In patients with transient ischaemic attack (TIA) and minor stroke, we assessed associations between hospital admissions with delirium and 5-year dementia risk and between admissions with infection and dementia risk, stratified by WMD severity (moderate or severe *vs* absent or mild) on baseline brain imaging.

**Methods:**

We included patients with TIA and minor stroke (National Institutes of Health Stroke Score <3) from the Oxford Vascular Study (OXVASC), a longitudinal population-based study of the incidence and outcomes of acute vascular events in a population of 94 567 individuals, with no age restrictions, attending eight general practices in Oxfordshire, UK. Hospitalisation data were obtained through linkage to the Oxford Cognitive Comorbidity, Frailty, and Ageing Research Database–Electronic Patient Records (ORCHARD-EPR). Brain imaging was done using CT and MRI, and WMD was prospectively graded according to the age-related white matter changes (ARWMC) scale and categorised into absent, mild, moderate, or severe WMD. Delirium and infection were defined by ICD-10 coding supplemented by hand-searching of hospital records. Dementia was diagnosed using clinical or cognitive assessment, medical records, and death certificates. Associations between hospitalisation with delirium and hospitalisation with infection, and post-event dementia were assessed using time-varying Cox analysis with multivariable adjustment, and all models were stratified by WMD severity.

**Findings:**

From April 1, 2002, to March 31, 2012, 1369 individuals were prospectively recruited into the study. Of 1369 patients (655 with TIA and 714 with minor stroke, mean age 72 [SD 13] years, 674 female and 695 male, and 364 with moderate or severe WMD), 209 (15%) developed dementia. Hospitalisation during follow-up occurred in 891 (65%) patients of whom 103 (12%) had at least one delirium episode and 236 (26%) had at least one infection episode. Hospitalisation without delirium or infection did not predict subsequent dementia (HR 1·01, 95% CI 0·86–1·20). In contrast, hospitalisation with delirium predicted subsequent dementia independently of infection in patients with and without WMD (2·64, 1·47–4·74; p=0·0013 *vs* 3·41, 1·91–6·09; p<0·0001) especially in those with unimpaired baseline cognition (cognitive test score above cutoff; 4·01, 2·23–7·19 *vs* 3·94, 1·95–7·93; both p≤0·0001). However, hospitalisation with infection only predicted dementia in those with moderate or severe WMD (1·75, 1·04–2·94 *vs* 0·68, 0·39–1·20; p_diff_=0·023).

**Interpretation:**

The increased risk of dementia after delirium is unrelated to the presence of WMD, whereas infection increases risk only in patients with WMD, suggesting differences in underlying mechanisms and in potential preventive strategies.

## Introduction

Episodes of delirium and infection are modifiable risk factors for dementia.^1–6^ Delirium is associated with an approximately two to three times greater risk of dementia (appendix pp 5, 6–8).^1^ Bacterial infections (eg, sepsis, pneumonia, urinary tract infection, and cellulitis) also increase the risk of dementia, with a dose-response relationship (number of infection episodes and severity of infection), largely independent of type of infection (appendix pp 5, 9–12).^3–6^ Delirium is thought to increase dementia through co-existent systemic inflammation caused by infection, other acute medical illness, or surgery.^7^ However, the independent effects of delirium and infection on dementia risk have not been widely studied despite their frequent co-existence (appendix pp 5, 6–12). In addition, associations between infection and subsequent dementia incidence might have been underestimated in previous studies through the use of routine diagnostic coding (ICD-10) to ascertain dementia in large administrative datasets. ICD-10 has limited sensitivity particularly for early versus advanced disease and sensitivity is further reduced by high rates of undiagnosed dementia.^4–6^

Cerebral white matter disease (WMD)^8,9^ and vascular-pattern cognitive impairment^10,11^ increase susceptibility to delirium. However, data on dementia incidence after delirium stratified by dementia subtype are sparse.^12^ In two studies examining dementia incidence by dementia subtype (defined using coded administrative data) after infection, the risk of vascular dementia was approximately two times greater than the risk of Alzheimer-type dementia (HR 4·15 *vs* 2·44 and HR 2·09 *vs* 1·20; appendix pp 5, 9–12).^4,5^ Delirium is also associated with subsequent longitudinal MRI changes in the periventricular, frontal, and temporal white matter,^13^ and sepsis can cause visible lesions on MRI.^12^ However, few studies of cognitive outcomes after delirium or infection used brain imaging and none used brain imaging to stratify analyses by the presence of WMD (appendix pp 5, 9–12). One study showed that atrophy related to Alzheimer’s disease, measured using MRI, was associated with increased risk of cognitive decline in patients with delirium.^14^ Regarding episodes of infection, two studies did not show changes in measures of WMD and atrophy over time after infection but power in these studies was low.^15,16^

WMD increases blood–brain barrier permeability and thereby cerebral vulnerability to systemic factors.^17^ Given the evidence of a link between cerebrovascular changes and delirium, and between infection and subsequent vascular dementia, we aimed to assess whether WMD on brain imaging would further increase susceptibility to dementia after delirium and infection. We therefore measured the associations between hospital admission with delirium after transient ischaemic attack (TIA) and minor stroke and 5-year risk of dementia, stratified by WMD severity (moderate or severe *vs* absent or mild) at baseline. We performed similar analyses to determine associations between hospitalisation for infection and subsequent dementia. We restricted analyses to TIA and minor stroke since the stroke lesion drives much of the post-stroke dementia risk after major events.^18,19^

## Methods

### Study design and population

We included patients with TIA and minor stroke (National Institutes of Health Stroke Score [NIHSS] <3) from the longitudinal population-based Oxford Vascular Study (OXVASC) and used hospitalisation data obtained through linkage to the Oxford Cognitive Comorbidity, Frailty, and Ageing Research Database-Electronic Patient Records (ORCHARD-EPR, figure). The study was undertaken and reported according to STROBE guidelines.

OXVASC is a population-based study of the incidence and outcomes of all acute vascular events in a population of 94 567 individuals, without age restrictions, registered with approximately 100 general practitioners in eight general practices in Oxfordshire, UK.^20,21^ The OXVASC population is 94% White, 3% Asian, 2% Chinese, and 1% Afro-Caribbean. Multiple overlapping methods of ascertainment allows for the detection of almost all events presenting to medical attention, therefore minimising selection bias.^21^ The study is approved by the regional ethics committee. Informed written consent was obtained from participants, or from relatives for patients lacking capacity to give informed consent to participate, for the study interviews and for the face-to-face follow-up interviews as well as for indirect follow-up using medical records. Consecutive patients with TIA or minor stroke were prospectively recruited from April 1, 2002, to March 31, 2012. Patients were assessed by a study clinician as soon as possible after their TIA or stroke. TIA was defined by symptoms, according to the National Institute of Neurological Disorders and Stroke criteria, and minor stroke was defined according to WHO criteria,^20,21^ with review of all cases as soon as possible after presentation by the same senior neurologist (PMR). Patients with major stroke (NIHSS ≥3) were excluded from analyses.

Patient data were collected via interview using a standardised form, supplemented by primary care records. Pre-morbid functional status was assessed with the modified Rankin Scale and stroke severity was defined using the NIHSS.^20,21^ The Charlson Comorbidity Index was calculated using data extracted from the study records according to updated UK weightings (appendix p 22).^22^ Cognition was assessed using the 10-point abbreviated mental test score (AMTS) or mini-mental state examination (MMSE). Baseline brain imaging was done predominantly with CT in the earlier years of the study and MRI in later years. WMD was prospectively graded by a neuroradiologist, and a subset of cases were also rated independently by an experienced neurologist as described previously (appendix p 15).^23^ Assessments were made blind to clinical data. WMD was graded according to the age-related white matter changes (ARWMC) scale developed for use in both CT and MRI.^24^ We used the total score derived from this scale to categorise patients as having absent, mild, moderate, or severe WMD (appendix p 15). We have previously shown that the ARWMC scale identifies moderate or severe WMD reliably on CT as well as on MRI.^23,24^

Multiple methods of follow-up were used to reduce attrition biases in identification of dementia, with follow-up to end of study or death completed in more than 95% of patients.^25,26^ Follow-up interviews were done by trained nurses or study physicians at 1 month, 6 months, 1 year, and 5 years. Cognitive function was assessed using the MMSE and the Montreal Cognitive Assessment (MoCA) for face-to-face interviews^27^ and the telephone version of the MoCA and the modified Telephone Interview of Cognitive Status, both of which have been validated for use in TIA and stroke, for telephone interviews.^28^

Dementia was defined as pre-event or post-event according to whether a diagnosis occurred before or after stroke or TIA, as described previously (appendix p 17).^19^ Briefly, a pre-event dementia diagnosis was made using the following information: (1) baseline clinical and cognitive assessment by a study physician and via discussions with informants; (2) dementia diagnosis, and related consultations and investigations, along with hand-searching of the entire primary care record, including individual consultations, clinic letters, hospitalisation documentation, and death certificates. Post-event dementia was diagnosed according to the same methods (ie, with baseline and follow-up clinical and cognitive assessment data, supplemented by hand-searching of death certificates and primary care records to death or 5-year follow-up). All dementia diagnoses were made using the DSM-IV criteria by a senior study physician with expertise in dementia (STP). Patients with pre-event dementia were excluded.

### Exposure to hospitalisation with delirium and infectious disease

For all patients, we supplemented OXVASC follow-up interviews by obtaining details of all elective and non-elective hospital admissions to all hospitals serving the study population (Oxford University Hospitals NHS Foundation Trust) through linkage of OXVASC data to ORCHARD-EPR which covers the entire OXVASC population (2002–12). ORCHARD-EPR contains pseudonymised individual hospital electronic patient records, including diagnoses based on the 10th revision of the International Statistical Classification of Diseases and Related Health Problems (ICD-10 codes).^29^ ORCHARD-EPR is approved by the regional ethics committee. For the purposes of this analysis, we excluded day cases, admissions before the index TIA and stroke event, and admissions occurring after the dementia diagnosis or 5 years after the index TIA or stroke.

Delirium was defined according to ICD-10-based diagnostic coding (appendix p19).^30^ In addition, the ICD-10 coded data were supplemented by hand-searching of medical records by two clinicians with expertise in delirium (AS, STP) who reviewed OXVASC study documentation and hospital case notes from the admission, where available, to check for evidence of delirium (appendix pp 19–21). Diagnosis of delirium was made by STP according to the Diagnostic and Statistical Model of Diseases, fourth edition (DSM-IV). We defined and classified hospital-treated infectious diseases according to ICD-10 hospital coding, and further classified bacterial infections to reflect systemic versus localised disease as per Sipilä and collagues.^4^

### Statistical analysis

Univariate analyses were undertaken comparing the clinical baseline characteristics between patients with no hospital admissions, patients hospitalised for infection or with delirium during the follow-up period, and those hospitalised for all other causes (ie, without delirium or infection).

We evaluated the associations between hospitalisation with delirium and hospitalisation for infection, and post-event dementia using Cox regression models with time-dependent covariates. All Cox models were stratified by WMD severity (moderate or severe *vs* absent or mild) on baseline brain imaging. The time origin for the Cox model was the date of the index TIA or minor stroke. Patients were censored at 5·5 years of follow-up, dementia diagnosis, or death, whichever happened first.

Regression analyses were adjusted first for baseline demographic variables (ie, age, sex, and education), and then for demographic variables and vascular risk factors and factors previously shown to be associated with dementia after TIA or stroke^18,19^ (ie, previous history of stroke, angina, myocardial infarction or peripheral arterial disease, treated hyperlipidaemia, treated hypertension, atrial fibrillation, pre-morbid modified Rankin scale score, level of education, smoking status, baseline cognitive score, event severity [as defined by NIHSS score], dysphasia at time of TIA or stroke event, and *APOE* genotype). In the fully adjusted model, both infection and delirium were entered together into the model so that their independent effects could be evaluated. For time-varying covariates, the time axis was divided into single day periods, in which the following were estimated: hospitalisation with delirium, hospitalisation with infection, or hospitalisation with neither; stroke recurrence; and the Charlson Comorbidity Index.^22^ For the Charlson Comorbidity Index, the baseline score was recalculated at each subsequent hospital admission depending on whether one or more of the 17 diseases in the Index was present according to the ICD-10 coded diagnoses (appendix p 22). The Cox proportional hazards assumption was assessed using Schoenfeld’s residuals. Analyses were repeated for different subtypes of infection (bacterial *vs* other and systemic *vs* localised bacterial infections), for those aged 80 years or older versus those younger than 80 years to determine whether associations were different in older patients, and analyses were also stratified by sex. Since data on infection severity or symptoms were not available, severity was proxied by the number of days in hospital with infection and analyses were repeated using days in hospital rather than admissions.

We performed a number of pre-specified sensitivity analyses. We assessed the associations of dementia with delirium (without infection in the model) and of dementia with infection (without delirium in the model). Given that dementia may predispose to infection^31^ and therefore that reverse causation might play a role in associations between infection and dementia, we performed a sensitivity analysis excluding all hospital admissions up to 1 year before dementia diagnosis. In addition, given that reduced baseline cognition is a strong predictor of future dementia risk, and to mitigate the possibility of reverse causation occurring through pre-event cognitive decline, we also restricted analyses to patients with unimpaired cognition at baseline (defined as AMTS ≥9 or MMSE ≥24). Given that baseline cognition data were missing for some patients, an ad-hoc sensitivity analysis was done by repeating the analyses without baseline cognition as a potential explanatory variable.

Due to the large number of covariates assessed in the regression model, analyses were also repeated using adaptive least absolute shrinkage and selection operator (lasso) regression analysis for model selection using the STATA “lasso cox” command with adaptive selection. Additionally, we assessed whether the results of our standard Cox regression models were biased by the presence of time-dependent confounders affected by previous admission with, or co-existence of, infection or delirium. In order to improve the estimation of the causal effect of infection and delirium on dementia, analyses were repeated using marginal structural models. We followed the approach used by Robins and colleagues^32^ and Fewell and coleagues^33^ by which we derived inverse-probability-of-exposure (separately for infection and delirium) weights, which were then used in a pooled logistic regression model to estimate the causal effect of exposure to infection and delirium on dementia.

All analyses were done in STATA/MP (version 18.0). Statistical significance was set at p<0·05.

### Role of the funding source

The funders of the study had no role in study design, data collection, data analysis, data interpretation, or writing of the report.

## Results

Of 2305 patients with TIA and stroke, 225 patients with pre-event dementia and 711 with major stroke were excluded. Of 1369 remaining patients (655 with TIA and 714 with minor stroke, mean age 72 [SD 13] years, 674 female and 695 male), 364 (27%) had moderate or severe WMD and 1005 (73%) had absent or mild WMD. During 4979 patient-years of follow-up (median 4·87 years), there was a total of 2212 admissions (1773 [80%] unplanned). At least one hospital admission occurred during follow-up in 891 (65%) patients, among whom 103 (12%) had at least one admission with delirium and 236 (26%) had at least one admission with infection. Patients with any admission with delirium were older than patients admitted for other reasons and patients not admitted to hospital (mean age 80 [SD 9] *vs* 72 [13] and 70 [13] years, p<0·0001) and had higher levels of vascular comorbidity, especially compared with patients without hospital admission, although differences attenuated after adjustment for age (table 1). Similarly, the 236 patients with infection were older and had more comorbidities than patients without infection (table 2).

Delirium occurred during 129 (6%) admissions, of which 45 (35%) had co-existent infection (appendix p 23). Infection occurred in 356 (16%) admissions. Almost all infections were bacterial (339 [95%]), of which 168 (50%) were systemic. Admissions associated with infection or delirium had longer lengths of stay (appendix p 23). Of the 1369 patients, 239 (17%) died during follow-up. During follow-up, 209 (15%) patients developed dementia. For the 41 (20%) patients who developed dementia after their first hospital admission with delirium, the median time from admission to dementia diagnosis was 0·44 (IQR 0·19–1·82) years (21 cases within 6 months after first delirium-related hospital admission, six between 6 months and 1 year, six between 1 and 2 years, and eight after at least 2 years). For the 35 (17%) patients who developed dementia after their first hospital admission with infection, the median time from admission to dementia diagnosis was 0·51 (0·19–1·16) years (15 cases within 6 months after first infection-related hospital admission, eight between 6 months and 1 year, five between 1 and 2 years, and seven after at least 2 years).

After adjustment for age and sex, delirium was associated with subsequent dementia in patients both with and without moderate or severe WMD (adjusted HR [aHR] 3·88, 95% CI 2·65–5·70; p<0·0001 and 1·78, 1·26–2·50; p=0·0001, table 3). Hospitalisation with infection was also associated with dementia but only in patients with moderate or severe WMD (aHR 1·95, 95% CI 1·34–2·84; p=0·0005) and not in those with absent or mild WMD (1·08, 0·73–1·59; p=0·70, p_diff_=0·0035).

In multivariable Cox-regression analysis, hospital-isation with delirium was associated with an increased risk of dementia independently of associated infection and other risk factors (aHR 2·69, 95% CI 1·82–3·97; p<0·0001), while hospitalisation without delirium or infection did not predict subsequent dementia risk (1·01, 0·86–1·20; p=0·87). This association was evident both in patients with and without WMD (aHR 2·64, 95% CI 1·47–4·74; p<0·0013 *vs* 3·41, 1·91–6·09; p<0·0001, table 3) and was strongest in older patients (7·64, 3·63–16·1; p<0·0001 and 3·09, 1·51–6·35; p=0·0021, for patients aged ≥80 years). In the same multivariable analysis, hospitalisation with infection remained predictive of dementia only in patients with moderate or severe WMD (aHR 1·75, 95% CI 1·04–2·94; p=0·037 *vs* 0·68, 0·39–1·20; p=0·19, p_diff_=0·023). The predictive value of infection for subsequent dementia in patients with moderate or severe WMD was driven mainly by patients younger than 80 years of age (aHR 13·3, 95% CI 1·70–104·0; p=0·013 *vs* 1·40, 0·75–2·61; p=0·30 for ≥80 years). There were no significant differences when stratified by sex (aHR 1·40, 95% CI 0·64–3·07; p=0·40 in females *vs* 1·80, 0·73–4·44; p=0·20 in males). There was little evidence of time-dependent confounding in our analyses, with results from the marginal structural models showing similar findings to the Cox regression models regarding the exposure effects for hospitalisation with delirium and with infection (appendix p 25).

Using a more parsimonious model as selected using lasso regression analysis (ie, after adjusting for age, education level, stroke severity, baseline cognition, and history of each of atrial fibrillation, angina, and myocardial infarction), hospitalisation with infection (aHR 1·68, 95% CI 1·05–2·69; p=0·036) and delirium (3·15, 1·98–5·01; p<0·0001) remained predictive of dementia in patients with moderate or severe WMD. For those with absent or mild WMD, the lasso analysis did not include hospitalisation with infection as a covariate in the model, and hospitalisation with delirium continued to be predictive of dementia (aHR 2·75, 95% CI 1·75–4·31; p<0·0001) after adjusting for age, gender, education level, baseline cognition, *APOE* genotype, and comorbidities.

Analyses were repeated after stratifying by bacterial infection and then further stratified by systemic versus localised bacterial infection. After multivariable adjustment, hospitalisation with bacterial infection was associated with an increased dementia risk in patients with moderate or severe WMD with aHR of 1·83 (95% CI 1·09–3·07; p=0·018 [systemic infection 2·23, 1·02–4·89; p=0·038 and localised infection 1·63, 0·89–2·97; p=0·11]) but not in patients with absent or mild WMD (0·69, 0·39–1·24; p=0·17; appendix pp 24,26). When using length of stay as a proxy of infection severity, we found an association between more days in hospital with infection and increased dementia risk in patients with moderate or severe WMD but not in those without moderate or severe WMD (aHR 1·04, 95% CI 1·01–1·08; p=0·0047 *vs* 1·01, 0·99–1·04; p=0·37).

In sensitivity analyses, after excluding all hospital admissions in the year before dementia diagnosis, the associations between hospitalisation with delirium and subsequent dementia were maintained in both patients with and without moderate or severe WMD (aHR 2·51, 95% CI 1·39–4·56; p=0·0022 and 3·46, 1·94–6·19; p<0·0001; table 4). After restricting the analysis to patients with unimpaired baseline cognition, associations between delirium and dementia were strengthened in both patients with and without WMD (aHR 3·94, 95% CI 1·95–7·93; p=0·0001 and 4·01, 2·23–7·19; p<0·0001; table 4). Similarly, associations were strengthened between hospitalisation with infection and dementia in patients with moderate or severe WMD (aHR 2·02, 95% CI 1·15–3·55; p=0·014).

## Discussion

Previous studies have shown that both delirium and infection increase dementia risk in a dose-dependent fashion^1–6^, with the measured effects possibly under-estimated owing to the frequent use of administrative diagnostic coding to ascertain dementia.^30^ In our study of dementia risk after TIA and minor stroke, we found that hospitalisation with delirium increased dementia risk irrespective of WMD over and above other measured predictors, including infection, and might therefore be a modifiable risk factor regardless of the underlying neuropathology. In contrast, infection was associated with an increased risk of dementia only in patients with moderate or severe WMD, suggesting an increased susceptibility conferred by cerebrovascular pathology.

The excess risk of dementia in patients with delirium in our study was similar in magnitude to that reported in previous studies (two to three times increased risk), despite longer follow-up and careful adjustment for baseline covariates, including baseline cognition, in our study (appendix p 5).^1^ However, this association between delirium and dementia risk was driven mainly by older (≥80 years) patients possibly because of the small number of younger patients with delirium. In addition, we showed that the effect of delirium on dementia risk was independent of infection. Infection is a frequent co-associate of delirium and is thought to be a key mediator in the association between delirium and increased dementia risk,^7^ but our study suggests that other mechanisms could also be important. Many delirium-related admissions (over half) occurred in the absence of any obvious infection, suggesting a role for non-infective mechanisms linked to other precipitants (eg, constipation, dehydration, metabolic derangement, medication, environmental factors, and occult acute cerebrovascular events).^7,34^ Future studies should aim to establish whether it is the delirium itself that increases dementia risk or whether the same factors drive both the delirium and future dementia risk.

Our results showed that delirium was associated with an increased dementia risk irrespective of the presence of WMD. There are few studies examining dementia risk after delirium according to neuroimaging findings or dementia subtype (appendix p 5).^1^ Delirium has been shown to alter the cognitive trajectory in patients with Alzheimer’s disease^35^ and to increase the risk of dementia after stroke,^18^ suggesting effects in individuals with neurodegenerative and or vascular pathology. This is supported by neuropathological studies suggesting that delirium increases dementia risk over and above the effects of measured neurodegenerative and vascular pathologies.^36^ Of note, we found that the increased risk of dementia was greatest in those with unimpaired baseline cognition, consistent with findings from population-based cohorts in which individuals with the highest pre-morbid cognitive function experienced the largest cognitive decrement after delirium,^2,37^ suggesting that delirium might even initiate neuropathological change.

The excess risk of dementia after infection in our study was similar to that reported in previous studies and also showed a dose-response effect (greater effects for systemic *vs* localised infection). The increased risk of dementia after infection was independent of delirium in the multivariable models and was driven by younger patients, consistent with one other study^38^ and in contrast to our findings of delirium increasing dementia risk predominantly in older individuals. However, this excess risk of dementia after infection was limited to patients with moderate or severe WMD, consistent with the two-times higher risk reported for vascular dementia versus Alzheimer’s disease in administrative, big data studies.^4,5^ As was the case with delirium, the increased risk of dementia after infection was greatest in those with unimpaired baseline cognition. WMD increases blood brain barrier permeability,^17^ facilitating entry of systemic inflammatory factors to the brain that might in turn promote inflammatory mechanisms linked to white matter damage and WMD progression.^7,39^ Neurodegenerative processes could also be affected since co-existent small vessel disease accelerates cognitive decline in Alzheimer’s disease.^40^

Strengths of our study include the longitudinal population-based design, ascertainment of dementia using study interview, mitigation of selection and attrition bias using multiple methods of follow-up, and adjustment for multiple covariates identified in previous studies. In addition, we were conservative in using two-sided tests of significance despite previous evidence linking delirium and infection to increased dementia risk. Limitations of our study include the partial reliance on administrative coding to ascertain delirium since it was not feasible to prospectively assess all patients during hospitalisation. Delirium coding is insensitive,^30^ but we supplemented coding by hand-searching available medical records. Residual under-ascertainment of delirium is possible, however, since the observed prevalence was lower than might be expected for older hospital in-patients.^34^ Measures of infection severity, symptoms, treatment, or antimicrobial use were unavailable in ORCHARD-EPR over this time period, but we used established methods to define localised and systemic infection and we used length of stay as a proxy for infection severity.^4^ We did sensitivity analyses to reduce the likelihood of reverse causation, but this could possibly still have explained some of the association between delirium and dementia. However, reverse causation is unlikely to have explained the association between infection and dementia since dementia has to be relatively severe before infection becomes likely.^31^ Finally, we assessed WMD using dicotomised WMD severity only as we were unable to capture the full extent of WMD owing to the use of CT-brain imaging in many patients, which might have underestimated the true WMD burden (although the sensitivity of CT and MRI for moderate or severe white matter change is similar).^23^

Our findings have a number of clinical implications. First, at least 30% of the population aged 75 years or older have WMD visible on brain imaging^41^ and brain imaging is frequently used in the course of routine care.^9^ Our study suggests that WMD quantification on routinely acquired brain scans could be used to inform risk–benefit calculations regarding the proactive use of antibiotics for early treatment of infection, prioritisation for vaccination, and counselling regarding the risks of elective surgery. In contrast, WMD burden does not appear to have relevance for measures to reduce the risk of dementia associated with delirium (although WMD indicates an increased risk of delirium). Second, the older hospitalised population is at high risk of vascular disease^30^ and at high risk of dementia overall, and therefore might be an ideal population from which to recruit to clinical trials of preventive strategies or treatments for dementia. The wide availability of prior routinely acquired brain imaging data would obviate the need for further imaging to identify WMD in trials of dementia prevention after infection. Patients with WMD but without cognitive impairment would be an important group to target since they are at high risk of dementia after infection but are not yet impaired.

In conclusion, WMD on brain imaging does not affect the increased risk of dementia after delirium and delirium could therefore be a modifiable risk factor for dementia irrespective of brain imaging findings. In contrast, the presence of WMD increases susceptibility to dementia after infection. Infection could therefore also be a modifiable risk factor for dementia but only in patients with WMD.

## Supplementary Material

Supplementary Appendix

## Figures and Tables

**Figure 1:**
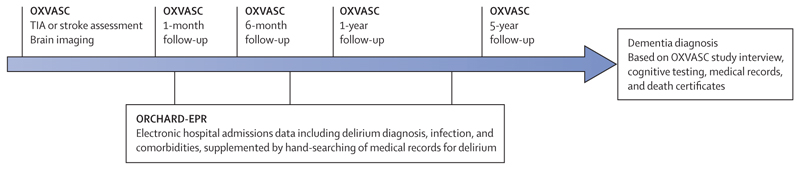
Schematic diagram showing the linkage of the longitudinal population-based OXVASC study of all TIA and stroke to the electronic hospitalisations data contained in the ORCHARD-EPR* ORCHARD-EPR=Oxford Cognitive Comorbidity, Frailty, and Ageing Research Database–Electronic Patient Records. OXVASC=Oxford Vasular Study. TIA=transient ischaemic attack. *Supplemented by hand-searching of medical records for evidence of delirium.

**Table 1:** Patient characteristics by hospitalisation status with and without delirium in 1369 transient ischaemic attack and minor stroke patients, adjusted for age and sex

	No hospital admissions (n=480)	Non-delirium-related hospital admissions (n=786)	Delirium-related hospital admissions (n=103)	No hospital admissions *vs* delirium-related hospital admissions, adjusted p value	Non-delirium-related hospital admissions *vs* delirium-related hospital admissions, adjusted p value
Age, years	70 (13)	72 (13)	80 (9)	NA	NA
Sex					
Male	237 (49%)	422 (54%)	34 (33%)	NA	NA
Female	243 (51%)	364 (46%)	69 (67%)		
Low education (<12 years)	297 (62%)	526 (67%)	70 (68%)	0·84	0·77
Pre-morbid disability	36 (8%)	89 (11%)	25 (24%)	0·027	0·16
Moderate or severe white matter disease	117 (26%)	205 (28%)	42 (42%)	0·22	0·38
Treated hypertension	248 (52%)	478 (61%)	72 (70%)	0·21	0·51
Diabetes	45 (9%)	128 (16%)	15 (15%)	0·18	0·95
Treated hyperlipidaemia	130 (27%)	267 (34%)	29 (28%)	0·76	0·46
Atrial fibrillation	51 (11%)	141 (18%)	21 (20%)	0·090	0·87
Coronary heart disease	60 (13%)	177 (23%)	20 (19%)	0·30	0·46
Peripheral arterial disease	21 (4%)	62 (8%)	10 (10%)	0·11	0·55
Previous stroke	27 (6%)	70 (9%)	14 (14%)	0·036	0·43
Current smoker	75 (16%)	114 (15%)	17 (17%)	0·0054	0·010
Low baseline cognitive score*	29 (7%)	67 (10%)	19 (21%)	0·0009	0·031
Minor stroke	202 (42%)	453 (58%)	55 (53%)	0·028	0·76
National Institutes for Health Stroke Scale, point	0·28 (0·61)	0·54 (0·81)	0·57 (0·84)	<0·0001	0·77
Dysphasia	8 (2%)	39 (5%)	11 (11%)	0·0009	0·023

Data are mean (SD) or n (%). Low baseline cognitive score defined as mini-mental state examination score of less than 24 or abbreviated mental test score of less than 9.

* Data were missing for 47 patients for no hospital admissions, 108 for non-delirium-related hospital admissions, and 14 for delirium-related hospital admissions.

**Table 2:** Patient characteristics by hospitalisation status with and without infection in 1369 transient ischaemic attack or minor stroke patients, adjusted for age and sex

	No hospital admissions (n=480)	Non-infection-related admissions (n=653)	Infection-related hospital admissions (n=236)	No hospital admissions *vs* infection-related hospital admissions, adjusted p value	Non-infection-related admissions *vs* infection-related admissions, adjusted p value
Age, years	70 (13)	72 (13)	77 (11)		
Sex					
Male	237 (49%)	351 (54%)	107 (45%)		
Female	243 (51%)	302 (46%)	129 (55%)		
Low education	297 (63%)	444 (68%)	155 (66%)	0·85	0·33
Pre-morbid disability	36 (8%)	67 (10%)	48 (20%)	0·0012	0·015
Moderate or severe white matter disease	117 (26%)	165 (27%)	82 (37%)	0·21	0·21
Treated hypertension	248 (52%)	393 (60%)	161 (68%)	0·014	0·21
Diabetes	45 (9%)	100 (15%)	43 (18%)	0·0004	0·15
Treated hyperlipidaemia	130 (27%)	222 (34%)	77 (33%)	0·16	0·98
Atrial fibrillation	51 (11%)	116 (18%)	47 (20%)	0·032	0·88
Coronary heart disease	60 (13%)	146 (22%)	53 (22%)	0·034	0·46
Peripheral arterial disease	21 (4%)	45 (7%)	27 (11%)	0·011	0·043
Previous stroke	27 (6%)	50 (8%)	35 (15%)	0·0011	0·010
Current smoker	75 (16%)	94 (14%)	37 (16%)	0·021	0·033
Low baseline cognitive score*	29 (7%)	60 (11%)	26 (13%)	0·082	0·95
Minor stroke	202 (42%)	376 (57%)	137 (58%)	<0·0001	0·51
National Institutes for Health Stroke Scale, point	0·28 (0·61)	0·55 (0·83)	0·56 (0·78)	<0·0001	0·86
Dysphasia	8 (2%)	35 (5%)	15 (6%)	0·008	0·58

Data are mean (SD) or n (%). Low baseline cognitive score defined as mini-mental state examination of less than 24 or abbreviated mental test score of less than 9.

* Data were missing for 48 patients for no hospital admissions, 94 for non-infection-related admissions, and 31 for infection-related hospital admissions.

**Table 3:** Associations between hospitalisations with infection or with delirium and risk of dementia stratified by baseline WMD on brain imaging

	Absent or mild WMD			Moderate or severe WMD	
	HR (95% CI)	p>(z)		HR (95% CI)	p>(z)
Age-adjusted and sex-adjusted models* (n=931, absent or mild WMD, and n=364, moderate or severe WMD)					
Infection only-related hospital admissions	1·08 (0·73–1·59)	0·70		1·95 (1·34–2·84)	0·0005
Delirium only-related hospital admissions	1·78 (1·26–2·50)	0·0001		3·88 (2·65–5·70)	<0·0001
All other admissions only	1·16 (1·00–1·35)	0·064		1·22 (1·08–1·39)	0·0021
Multivariable adjusted model† (n=793, absent or mild WMD, and n=299, moderate or severe WMD)					
Infection-related hospital admissions	0·68 (0·39–1·20)	0·19		1·75 (1·04–2·94)	0·037
Delirium-related hospital admissions	3·41 (1·91–6·09)	<0·0001		2·64 (1·47–4·74)	0·0013
All other admissions	0·99 (0·78–1·27)	0·97		1·07 (0·86–1·35)	0·53

WMD=white matter disease.

* In the models adjusted for age and sex, infection or delirium or other admissions were each included in separate models.

† Multivariable analyses adjusted for: age; gender; previous history of angina, myocardial infarction, treated hyperlipidaemia, treated hypertension, atrial fibrillation, and peripheral arterial disease; pre-morbid disability (pre-morbid modified Rankin Scale score >2); level of education (≤12 years); smoking status; baseline cognitive score; event severity (National Institutes for Health Stroke Scale score); dysphasia at time of transient ischaemic attack or stroke event; *APOE* genotype; recurrent stroke with infection, delirium and all other admissions entered into the same model.

**Table 4:** Multivariable adjusted* associations between hospitalisations with infection or with delirium and risk of dementia stratified by WMD severity on baseline brain imaging in specified sensitivity analyses including both infection and delirium and all other admissions in the model

	Absent or mild WMD (n=793)			Moderate or severe WMD (n=299)	
	HR (95% CI)	P>(z)		HR (95% CI)	P>(z)
Main analysis					
Infection-related hospital admission	0·68 (0·39–1·20)	0·19		1·75 (1·04–2·94)	0·037
Delirium-related hospital admission	3·41 (1·91–6·09)	<0·0001		2·64 (1·47–4·74)	0·0013
All other admissions	0·99 (0·78–1·27)	0·97		1·07 (0·86–1·35)	0·53
Delirium not included					
Infection-related hospital admission	070 (0·41–1·19)	0·19		2·26 (1·38–3·69)	0·0013
All other admissions	1·10 (0·89–1·37)	0·39		1·13 (0·93–1·38)	0·22
Infection not included					
Delirium-related hospital admission	3·29 (1·85–5·85)	0·0001		3·23 (1·86–5·59)	<0·0001
All other admissions	0·94 (0·76–1·17)	0·59		1·11 (0·91–1·36)	0·30
Excluding admissions occurring within 1 year of dementia diagnosis					
Infection-related hospital admission	0·66 (0·36–1·19)	0·17		1·73 (1·02–2·95)	0·043
Delirium-related hospital admission	3·46 (1·94–6·19)	<0·0001		2·51 (1·39–4·56)	0·0022
All other admissions	1·00 (0·78–1·29)	0·98		1·07 (0·85–1·36)	0·57
Baseline cognition not included (n=849, absent or mild WMD, and n=309, moderate or severe WMD)					
Infection-related hospital admission	0·78 (0·47–1·27)	0·31		1·74 (1·05–2·89)	0·032
Delirium-related hospital admission	3·03 (1·73–5·32)	0·0004		2·90 (1·65–5·09)	0·0002
All other admissions	1·03 (0·89–1·37)	0·38		1·11 (0·90–1·36)	0·32
Patients with unimpaired cognition (n=739, absent or mild WMD, and n= 260, moderate or severe WMD)					
Infection-related hospital admission	1·03 (0·54–1·98)	0·92		2·02 (1·15–3·55)	0·014
Delirium-related hospital admission	4·01 (2·23–7·19)	<0·0001		3·94 (1·95–7·93)	0·0001
All other admissions	0·91 (0·67–1·24)	0·54		1·11 (0·87–1·41)	0·42

WMD=white matter disease.

* Multivariable analyses adjusted for: age; gender; previous history of angina, myocardial infarction, treated hyperlipidaemia, treated hypertension, atrial fibrillation, and peripheral arterial disease; pre-morbid disability (pre-morbid modified Rankin scale score >2); level of education (≤12 years); smoking status; baseline cognitive score; event severity (National Institutes for Health Stroke Scale score); dysphasia at time of transient ischaemic attack or stroke event; *APOE* genotype; recurrent stroke.

## Data Availability

Requests for access to OXVASC data should be made to Prof Peter Rothwell at peter.rothwell@ndcn.ox.ac.uk. Requests for access to ORCHARD-EPR data should be made to Prof Sarah Pendlebury at sarah.pendlebury@ndcn.ox.ac.uk.

## Research in context

### Evidence before this study

We updated previously published systematic reviews on delirium and infection, and future dementia risk by searching MEDLINE and Embase from Jan 1, 2019, (the end date of the previous searches) to Nov 27, 2023. For delirium, search terms included “delirium” and “dementia” or “cognitive decline/cognitive dysfunction” or “memory” or “memory disorders” or “episodic memory”. For infection, search terms included “pneumonia” or “lower respiratory tract infection” or “cystitis” or “cellulitis” or “sepsis” or “systemic inflammatory response syndrome” and the same terms for dementia and cognitive decline as used for the delirium searches. Synthesis of the evidence shows that both delirium and infection are risk factors for dementia, with dose-response effects. Findings were similar in studies (delirium=13 studies, infection=16 studies) published since the previous systematic reviews (delirium pooled OR 2·30, 95% CI 1·85–2·86, n=24 studies; bacterial infection HR 1·10, 95% CI 1·02–1·19 to OR 2·60; 1·84–3·66, n=19 studies). Infection was associated with a relatively greater risk of vascular versus Alzheimer-type dementia (HR 4·15 *vs* 2·44 and 2·09 *vs* 1·20 in two studies) but data stratified by dementia subtype for delirium were lacking. Although delirium and infection frequently co-exist, only one study considered both conditions together and none of the studies included baseline brain imaging or stratified findings by presence of cerebral white matter disease (WMD).

### Added value of this study

We found that in patients with previous transient ischaemic attack or minor stroke, both delirium and infection increased the risk of subsequent dementia, in line with previous studies. However, we filled an important evidence gap in showing that the effects of delirium and infection on dementia risk were independent of one another. Furthermore, the effect of delirium on dementia risk was greater in older patients(≥80 years), whereas the impact of infection was greater in younger patients (<80 years). Most notably, we found that the presence of WMD on baseline brain imaging modified dementia risk associations: infection only increased dementia risk in those patients with WMD, whereas delirium increased the risk irrespective of the presence of WMD.

### Implications of all the available evidence

Delirium and infection are independent and potentially modifiable risk factors for dementia. The longer-term cognitive effects of delirium might therefore not be solely mediated by co-existent infection and the cognitive effects of infection might not be solely mediated by co-existent delirium. This, together with the risk modifying effects of WMD, suggests that there are different mechanisms underpinning dementia occurring after infection versus after delirium, and further studies are required to inform future preventive treatments. Dementia prevention after infection should be focused especially on those with WMD, with the potential to reduce vascular dementia in particular.

## References

[1] Goldberg TE, Chen C, Wang Y (2020). Association of delirium with long-term cognitive decline: a meta-analysis. JAMA Neurol.

[2] Tsui A, Searle SD, Bowden H (2022). The effect of baseline cognition and delirium on long-term cognitive impairment and mortality: a prospective population-based study. Lancet Healthy Longev.

[3] Muzambi R, Bhaskaran K, Brayne C, Davidson JA, Smeeth L, Warren-Gash C (2020). Common bacterial infections and risk of dementia or cognitive decline: a systematic review. J Alzheimers Dis.

[4] Sipilä PN, Heikkilä N, Lindbohm JV (2021). Hospital-treated infectious diseases and the risk of dementia: a large, multicohort, observational study with a replication cohort. Lancet Infect Dis.

[5] Chu CS, Liang CS, Tsai SJ (2022). Bacterial pneumonia and subsequent dementia risk: a nationwide cohort study. Brain Behav Immun.

[6] Morton CE, Forbes HJ, Pearce N, Smeeth L, Warren-Gash C (2020). Association between common infections and incident post-stroke dementia: a cohort study using the clinical practice research datalink. Clin Epidemiol.

[7] Wilson JE, Mart MF, Cunningham C (2020). Delirium. Nat Rev Dis Primers.

[8] Clancy U, Gilmartin D, Jochems ACC, Knox L, Doubal FN, Wardlaw JM (2021). Neuropsychiatric symptoms associated with cerebral small vessel disease: a systematic review and meta-analysis. Lancet Psychiatry.

[9] Pendlebury ST, Thomson RJ, Welch SJV, Kuker W, Rothwell PM (2022). Utility of white matter disease and atrophy on routinely acquired brain imaging for prediction of long-term delirium risk: population-based cohort study. Age Ageing.

[10] Hasegawa N, Hashimoto M, Yuuki S (2013). Prevalence of delirium among outpatients with dementia. Int Psychogeriatr.

[11] Pendlebury ST, Thomson RJ, Welch SJV, Rothwell PM (2022). Cognitive predictors of delirium on long-term follow-up after TIA and stroke: population-based cohort study. Cerebrovasc Dis.

[12] Sharshar T, Carlier R, Bernard F (2007). Brain lesions in septic shock: a magnetic resonance imaging study. Intensive Care Med.

[13] Cavallari M, Dai W, Guttmann CRG (2017). Longitudinal diffusion changes following postoperative delirium in older people without dementia. Neurology.

[14] Racine AM, Touroutoglou A, Abrantes T (2020). Older patients with Alzheimer’s disease-related cortical atrophy who develop post-operative delirium may be at increased risk of long-term cognitive decline after surgery. J Alzheimers Dis.

[15] Muzambi R, Bhaskaran K, Rentsch CT (2022). Are infections associated with cognitive decline and neuroimaging outcomes? A historical cohort study using data from the UK Biobank study linked to electronic health records. Transl Psychiatry.

[16] Peters van Ton AM, Meijer-van Leijsen EMC, Bergkamp MI (2022). Risk of dementia and structural brain changes following non-neurological infections during 9-year follow-up. Crit Care Med.

[17] Walsh J, Tozer DJ, Sari H (2021). Microglial activation and blood-brain barrier permeability in cerebral small vessel disease. Brain.

[18] Pendlebury ST, Rothwell PM (2009). Prevalence, incidence, and factors associated with pre-stroke and post-stroke dementia: a systematic review and meta-analysis. Lancet Neurol.

[19] Pendlebury ST, Rothwell PM (2019). Incidence and prevalence of dementia associated with transient ischaemic attack and stroke: analysis of the population-based Oxford Vascular Study. Lancet Neurol.

[20] Rothwell PM, Coull AJ, Silver LE (2005). Population-based study of event-rate, incidence, case fatality, and mortality for all acute vascular events in all arterial territories (Oxford Vascular Study). Lancet.

[21] Coull AJ, Silver LE, Bull LM, Giles MF, Rothwell PM (2004). Direct assessment of completeness of ascertainment in a stroke incidence study. Stroke.

[22] Charlson M, Szatrowski TP, Peterson J, Gold J (1994). Validation of a combined comorbidity index. J Clin Epidemiol.

[23] Simoni M, Li L, Paul NL (2012). Age- and sex-specific rates of leukoaraiosis in TIA and stroke patients: population-based study. Neurology.

[24] Wahlund LO, Barkhof F, Fazekas F (2001). A new rating scale for age-related white matter changes applicable to MRI and CT. Stroke.

[25] Pendlebury ST, Chen PJ, Bull L, Silver L, Mehta Z, Rothwell PM (2015). Methodological factors in determining rates of dementia in transient ischemic attack and stroke: (I) impact of baseline selection bias. Stroke.

[26] Pendlebury ST, Chen PJ, Welch SJ (2015). Methodological factors in determining risk of dementia after transient ischemic attack and stroke: (II) effect of attrition on follow-up. Stroke.

[27] Pendlebury ST, Mariz J, Bull L, Mehta Z, Rothwell PM (2012). MoCA, ACE-R, and MMSE versus the National Institute of Neurological Disorders and Stroke-Canadian Stroke Network Vascular Cognitive Impairment Harmonization Standards Neuropsychological Battery after TIA and stroke. Stroke.

[28] Pendlebury ST, Welch SJ, Cuthbertson FC, Mariz J, Mehta Z, Rothwell PM (2013). Telephone assessment of cognition after TIA and stroke: TICSm and telephone MoCA vs face-to-face MoCA and neuropsychological battery. Stroke.

[29] Boucher E, Jell A, Singh S (2023). The Oxford Cognitive Comorbidity and Ageing Research Database (ORCHARD): description of a large acute care research database. Research Square.

[30] Pendlebury ST, Lovett NG, Thomson RJ, Smith SC (2020). Impact of a system-wide multicomponent intervention on administrative diagnostic coding for delirium and other cognitive frailty syndromes: observational prospective study. Clin Med (Lond).

[31] Shah FA, Pike F, Alvarez K (2013). Bidirectional relationship between cognitive function and pneumonia. Am J Respir Crit Care Med.

[32] Robins JM, Hernán MA, Brumback B (2000). Marginal structural models and causal inference in epidemiology. Epidemiology.

[33] Fewell Z, Wolfe F, Choi H, Hernan MA, Tilling K, Sterne JAC (2004). Controlling for time-dependent confounding using marginal structural models. Stata J.

[34] Pendlebury ST, Lovett NG, Smith SC (2015). Observational, longitudinal study of delirium in consecutive unselected acute medical admissions: age-specific rates and associated factors, mortality and re-admission. BMJ Open.

[35] Fong TG, Jones RN, Shi P (2009). Delirium accelerates cognitive decline in Alzheimer disease. Neurology.

[36] Davis DH, Muniz-Terrera G, Keage HA (2017). Association of delirium with cognitive decline in late life: a neuropathologic study of 3 population-based cohort studies. JAMA Psychiatry.

[37] Krogseth M, Davis D, Jackson TA (2023). Delirium, neurofilament light chain, and progressive cognitive impairment: analysis of a prospective Norwegian population-based cohort. Lancet Healthy Longev.

[38] Chalitsios CV, Baskaran V, Harwood RH, Lim WS, McKeever TM (2023). Incidence of cognitive impairment and dementia after hospitalisation for pneumonia: a UK population-based matched cohort study. ERJ Open Res.

[39] Low A, Mak E, Rowe JB, Markus HS, O’Brien JT (2019). Inflammation and cerebral small vessel disease: a systematic review. Ageing Res Rev.

[40] Tosto G, Zimmerman ME, Hamilton JL, Carmichael OT, Brickman AM (2015). The effect of white matter hyperintensities on neurodegeneration in mild cognitive impairment. Alzheimers Dement.

[41] Mok V, Srikanth V, Xiong Y (2014). Race-ethnicity and cerebral small vessel disease—comparison between Chinese and White populations. Int J Stroke.

