# Supplementary material for: Infection, delirium, and risk of dementia in patients with and without white matter disease on previous brain imaging: a population-based study
Source: Lancet Healthy Longev. Author manuscript; Available in PMC 2025 Aug 14. (PMC7618011; doi:10.1016/S2666-7568(23)00266-0)

THE LANCET
Healthy Longevity

Supplementary appendix

This appendix formed part of the original submission and has been peer reviewed. We post it as supplied by the authors.

Supplement to: Pendlebury ST, Luengo-Fernandez R, Seeley A, et al. Infection, delirium, and risk of dementia in patients with and without white matter disease on previous brain imaging: a population-based study. *Lancet Healthy Longev* 2024; **5:** e131–40.

Online supplementary material

Index

Updated systematic reviews, Methods 1-4

Systematic reviews, Results 5-12

Table S1 6

Table S2 9

References 13

Brain imaging and rating of white matter disease (WMD) on CT and MRI brain scans 15

Figure S1. Brain images showing moderate/severe WMD 16

Dementia diagnosis 17

Delirium ascertainment 19

Table S3. Charlson index 22

Table S4. Hospital admissions on 5-year follow up 23

Table S5. Bacterial infection and risk of dementia 24

Figure S2. Marginal Structural Models 25

Figure S3. Systemic vs localised infection and dementia 26

Supplemental Methods

Updated systematic review on delirium and risk of dementia or cognitive decline.

The systematic review by Goldberg et al[1] on delirium and risk of dementia or cognitive decline reported a pooled OR for dementia after delirium of 2.30, 95%CI 1.85-2.86, across 24 studies. We updated this systematic review by searching Medline and Embase using a similar search strategy as described in the previous review (see details below) from the end date of the previous search (1st January 2019) to 27th November 2023. We included longitudinal studies of adults aged >/=18 years where either dementia or cognitive decline measured using validated methods (dementia diagnosis from assessments or medical records, cognitive testing) was ascertained on follow-up, included a control/comparator group, and where delirium preceded dementia diagnosis. We excluded studies on individuals aged <18 years, that used proxy measures for delirium eg antipsychotic prescription, or used telephone interview without a cognitive test or medical records review to ascertain cognitive decline, or used cross-sectional study design or with sample size<100. After exclusion of duplicates and non-relevant titles/articles, we found 13 articles that met inclusion criteria (see Table S1)

Updated systematic review on common bacterial infections and risk of dementia or cognitive decline.

The systematic review by Muzambi et al[2] included 19 studies on common bacterial infections and risk of dementia or cognitive decline and reported a pooled risk of dementia of HR=1.10; 95% CI 1.02-1.19 and OR=2.60; 1.84-3.66. We updated this systematic review by searching Medline and Embase using a similar search strategy as described in the previous review (see details below) from the end date of the previous search (2st March 2019) to 27th November 2023. We included longitudinal studies of adults aged >/=18 years where either dementia or cognitive decline measured using validated methods was ascertained on follow-up, included a control/comparator group, and where infection preceded dementia diagnosis. We excluded studies on individuals aged <18 years, that examined non-bacterial infection including covid-19, used cross-sectional study design, or considered infection occurring after dementia diagnosis or with sample size<100. After exclusion of duplicates and non-relevant titles/articles, we found 16 articles that met inclusion criteria (see Table S2)

Literature Searches: details of searches

Delirium and dementia

Database:
Medline (Ovid MEDLINE® Epub Ahead of Print, In-Process & Other Non-Indexed Citations, Ovid MEDLINE® Daily and Ovid MEDLINE®) 1946 to present

#	Query	Results from 27 Nov 2023
1	*Emergence Delirium/ or delirium.mp. or *Delirium/	24,568
2	dementia.mp. or *Dementia/ or *"Mental Status and Dementia Tests"/ or *Dementia, Vascular/ or *Dementia, Multi-Infarct/	163,470

3	cognitive decline.mp. or *Cognitive Dysfunction/	59,031
4	*Memory/ or *Memory Disorders/ or *Memory, Short-Term/ or *Immunological Memory Cells/ or memory.mp. or *Memory, Episodic/ or *Wechsler Memory Scale/	351,677
5	cognition disorders.mp. or *Cognition Disorders/ or *Cognitive Dysfunction/	98,958
6	2 or 3 or 4 or 5	553,234
7	1 and 6	4,861
8	limit 7 to (english language and humans and yr="2019 -Current")	1,159

Database:
Embase 1974 to present

#	Query	Results from 27 Nov 2023
1	*hyperactive delirium/ or *delirium/ or *postoperative delirium/ or delirium.mp. or *hypoactive delirium/	47,828
2	*multiinfarct dementia/ or *dementia/ or *mixed dementia/ or dementia.mp. or *"mixed depression and dementia"/	256,192
3	cognitive deficit.mp. or *cognitive defect/	73,679
4	*cognitive defect/ or *cognition/ or cognitive decline.mp. or *Alzheimer disease/	317,868
5	*short term memory/ or *memory disorder/ or *Wechsler memory scale/ or *verbal memory/ or *episodic memory/ or *memory/ or *word memory test/ or memory.mp.	497,971
6	postoperative cognitive dysfunction.mp. or *postoperative cognitive dysfunction/ or *benzodiazepine derivative/	14,879
7	2 or 3 or 4 or 5 or 6	895,187
8	1 and 7	11,258
9	limit 8 to (human and english language and yr="2019 -Current")	3,563

Infection and dementia

Database:
Medline (Ovid MEDLINE® Epub Ahead of Print, In-Process & Other Non-Indexed Citations, Ovid MEDLINE® Daily and Ovid MEDLINE®) 1946 to present

#	Query	Results from 27 Nov 2023
1	*Pneumonia/ or pneumonia.mp. or *Pneumonia, Bacterial/	230,966
2	*Pneumonia/ or *Respiratory Tract Infections/ or lower respiratory tract infection.mp. or *Adult/	72,785
3	urinary tract infection.mp. or *Urinary Tract Infections/	47,755
4	*Cystitis/ or cystitis.mp.	16,375
5	*Cellulitis/ or cellulitis.mp.	15,118
6	*Sepsis/ or sepsis.mp.	153,217
7	systemic inflammatory response syndrome.mp. or *Systemic Inflammatory Response Syndrome/ or *Infections/	34,296
8	1 or 2 or 3 or 4 or 5 or 6 or 7	497,350
9	dementia.mp. or *Dementia/ or *"Mental Status and Dementia Tests"/ or *Dementia, Vascular/ or *Dementia, Multi-Infarct/	163,470
10	cognitive decline.mp. or *Cognitive Dysfunction/	59,031
11	*Memory, Long-Term/ or *Memory, Short-Term/ or memory.mp. or *Wechsler Memory Scale/ or *Memory Disorders/ or *Memory/ or *Immunological Memory Cells/ or *Memory, Episodic/	351,677
12	cognition disorders.mp. or *Cognition Disorders/ or *Cognitive Dysfunction/	98,958
13	9 or 10 or 11 or 12	553,234
14	8 and 13	3,498
15	limit 14 to (english language and humans and yr="2019 -Current")	992

Database:
Embase 1974 to present

#	Query	Results from 27 Nov 2023
1	*pneumococcal pneumonia/ or *infectious pneumonia/ or *community acquired pneumonia/ or *Staphylococcus aureus pneumonia/ or *multilobar pneumonia/ or pneumonia.mp. or	417,241

	*bilateral pneumonia/ or *lobar pneumonia/ or *bacterial pneumonia/ or *health care associated pneumonia/ or *Streptococcus pneumonia/ or pneumonia/ or *staphylococcal pneumonia/ or *hospital acquired pneumonia/	
2	lower respiratory tract infection.mp. or *lower respiratory tract infection/	17,586
3	urinary tract infection.mp. or *urinary tract infection/	137,384
4	*cystitis/ or cystitis.mp.	32,870
5	cellulitis.mp. or *cellulitis/	31,407
6	sepsis.mp. or *Gram positive sepsis/ or *sepsis/ or *Gram negative sepsis/	273,565
7	systemic inflammatory response syndrome.mp. or *systemic inflammatory response syndrome/	19,611
8	1 or 2 or 3 or 4 or 5 or 6 or 7	829,299
9	*multiinfarct dementia/ or *dementia/ or *mixed dementia/ or dementia.mp. or *senile dementia/	256,192
10	cognitive dysfunction.mp. or *cognitive defect/	90,918
11	*short term memory/ or *memory disorder/ or *Wechsler memory scale/ or *verbal memory/ or *episodic memory/ or memory.mp. or memory/	497,971
12	cognitive decline.mp.	51,011
13	*cohort analysis/ or cohort.mp.	1,637,844
14	9 or 10 or 11 or 12	769,290
15	8 and 13 and 14	1,441
16	limit 15 to (human and english language and yr="2019 -Current")	758

Systematic Review Results

Dementia after delirium

From 2019 onwards (since the previous systematic review), we identified 13 studies of dementia or cognitive decline after delirium (four with dementia outcomes and ten with measured cognitive change over time or cognitive impairment, Table S1).[3-15] In contrast to the infection studies, all but two studies were prospective in design probably because of the known very low sensitivity of hospital administrative ICD-10 coded data for delirium, and also to a lesser extent for dementia and therefore the possible confounding by undiagnosed pre-admission dementia.[16] Four out of four studies measuring dementia outcomes showed associations between delirium and subsequent dementia with evidence of a dose responsive effect.[3,4,11,14] All ten studies showed associations with worse cognitive function or deterioration in cognitive function over time but in one study this was only apparent in those with cortical thinning in a pattern suggestive of pre-existing Alzheimer-type pathology.[13] No studies stratified risk of dementia by baseline brain imaging findings or examined the risks by the different dementia subtypes. No study adjusted for co-existent infection.

Dementia after infection

From 2019 onwards (since the previous systematic review), we identified 16 studies of dementia or cognitive decline after infection (12 with dementia outcomes and five with measured cognitive change over time, Table S2).[17-32] Only 5/16 studies were prospective with the majority being retrospective “Big Data” studies in which dementia diagnosis was necessarily based on ICD-10 diagnostic administrative coding. Ten of the 12 studies with dementia outcomes showed a positive association with dementia which appeared to be dose responsive ie risk greater with repeated infection or severe infection defined as associated with the systemic inflammatory response syndrome (SIRS), intensive care unit stay, systemic vs localised infection, hospitalisation or measures of sepsis.[18,20,23,26,30] In one study, the associations were found to be stronger in younger vs older individuals.[19] Only one study adjusted for co-existent delirium.[22] Two studies examined the risk by dementia subtype finding higher risks (two- fold) for vascular vs Alzheimer dementia.[20,30] One study found that the increased risk of dementia after infection was higher in those with diabetes in whom cerebrovascular disease is known to be prevalent.[27] No study stratified the risk according to baseline brain imaging findings. The two studies that included brain imaging (MRI) found no associations between infection, structural brain scan abnormalities, and cognitive decline, and no change in brain scan changes over time after infection but both studies had high rates of selection and attrition on follow-up that may have contributed to the null results.[28,29]

Table S1: Associations between delirium and dementia or cognitive decline in studies published from April 2019 until 31st December 2022.

Study	N, Setting	Study design	Age, Sex	Delirium ascertainment	Brain imaging	Outcome measure	Findings
Bayer et al, 2022, USA[3]	14,385 residents hospitalised with heart failure	Retrospective	Mean age 76 years; 97% male, 15.7% Black)	CAM, routinely acquired in minimum admission dataset	N	Alzheimer's disease and related dementias via VA and medicare claims	adjusted HR=1.9, 95%CI 1.6, 2.3).
Chu et al, 2022, USA[4]	894 kidney transplant recipients	Prospective	Age ≥18 years	Chart review	N	FU 2009-2015 Dementia in Medicare claims data<br>Trail making test	adjusted subdistribution HR=7.84, 95%CI: 1.2-50.4
Giroux et al, 2022 Quebec, Canada[5]	393 ED patients	Prospective	Age ≥65 years	CAM	N	TICSm at baseline and 60 days	TICSm change =-1.6 (delirious) vs 0.5 (non-delirious), p=0.03
Hayhurst et al, 2022, USA[6]	502 ICU survivors of respiratory failure or shock	Prospective	Median (IQR) age 61 (52–70) years	CAM-ICU<br>Richmond Agitation and Sedation Scale	N	Repeatable Battery for Assessment of Neurological Function<br>Trail making test at 3 and 12 months	Hypo- but not hyper-active delirium was associated with global cognitive function at 3 months and executive function at 3 and 12 months
Jaatinen et al, 2021, Finland[7]	476 patients with hip fracture and without known pre-admission cognitive disorder	Prospective	Age ≥65 years	CAM	N	New cognitive disorder by interview and records review at 1 year	AdjOR=2.29 (1.39-3.79)
Knaak et al, 2020, Germany[8]	535 elective surgical patients	Prospective	Media (IQR) age 71 (67-76) with pre-surgical cognitive impairment and 69 (65-	DSM 4 criteria	N	Pre-operative cognitive impairment (2SD below controls).<br>Post-operative cognitive disorder by the reliable change index at 3 months	PreCI+delirium more likely to develop POCD [adjOR 6.131 (95%CI 1.476 to 22.364); P=0.007].

			74) in those without				
Krogseth et al, 2023, Norway[9]	203 community dwelling, receiving care ≥once weekly	Prospective	Age>65 years	DSM 5 criteria	N	Cognitive decline defined by change in MoCA score. Follow-up to 2 years.	Adjusted MoCA mean difference -1.5 (95%CI -2.9 to -0.1) over 6 months
Kunicki et al, 2023, USA[10]	560 community dwelling older adults	Prospective	Age >70 years	CAM and medical records review	N	Change in general cognitive performance (GCP)	Participants with delirium showed faster long-term cognitive change with an additional -0.4 GCP units (95% CI, -0.1 to -0.7) or -1.4 units per year.
Leighton et al, 2022, UK[11]	12, 949 Safe Haven database	Retrospective	Age ≥65 years	ICD-10 administrative diagnostic coding	N	Dementia ICD-10 administrative diagnostic coding at mean 741 days	HR for dementia increased with increasing age and deprivation
Nerdal et al, 2022, Norway[12]	139 acute stroke	Prospective	Mean (SD) age 71.4 (13.4) years	CAM	N	MoCA to 36 months	Delirious patients had lower MoCA at 18 months (Mean (SE): 20.8 (1.4) versus (25.1 (0.4)
Racine et al, 2020, USA[13]	Surgical patients	Prospective	Age ≥70 years	CAM	Y, MRI	Neuropsychological battery at baseline; months 1, 2, and 6; and every 6 months thereafter until 36 months.	Patients with delirium who had thinner AD signature cortex showed greater decline on a verbal learning test (beta = -0.100 [-0.192, -0.007]
Richardson et al, 2021, UK[14]	205 (173 with outcome data) Participants in the longitudinal Cognitive Function and Ageing Study study (CFAS)	Prospective	Age ≥65 years	DSM 5 criteria	N	MMSE<br>Dementia by prospective clinical assessment	Delirium was associated with cognitive decline (-1.8 Mini-Mental State Examination points [95% CI -3.5 to -0.2]) Dementia adj OR=8.8 [95% CI 1.9-41.4]). More than one episode and more days with delirium (>5 days) were associated with worse cognitive outcomes.

Webber et al, 2023, Canada[15]	92,005 long term care residents	Retrospective	Age ≥65 years Mean/SD 85.6 /8.2 years	Probable delirium defined by the delirium Clinical Assessment Protocol, part of the Resident Assessment Instrument-Minimum Dataset	N	Cognitive decline as defined by the Cognitive Performance Scale (range 0–6, higher values indicate greater impairment)	Adjusted ORs for cognitive decline of 1.64 (95%CI 1.35–1.99), 1.56 1.34–1.85), 1.57, 1.32–1.86) and 1.50, 1.25–1.80) after 1–3, 4–6, 7–9, and 10–12 months of follow-up

Table S2: Associations between infection and dementia or cognitive decline in studies published from April 2019 until 31st December 2022.

Study	N, Setting	Study design	Age	Infection	Brain imaging	Outcome measure	Findings
Ahlstrom et al, 2020, Sweden[17]	210,334 dementia free at 1-year Electronic records including Swedish dementia registry ICU	Retrospective	Median (IQR)=61 (43-72) years	Sepsis	N	Dementia via FU=11 years, median 3.9 years	Adjusted (HR) 1.01 (95% confidence interval 0.91-1.11, p = 0.873) Age, increasing severity of acute illness were strong risk factors for dementia.
Bohn et al, 2023, USA[18]	15,688 in the Atherosclerosis Risk in Communities (ARIC) study	Prospective	Mean/SD baseline age, 54.7/5.8 years	Hospitalizations with infection identified via medical record review	N	Incident dementia identified through ICD-9 and ICD-10 hospitalization and death certificate codes, in-person assessments, and telephone interviews.	Unadjusted HR=2.02 (95% CI, 1.88-2.18; P < .001); adjusted HR=1.70 (95% CI, 1.55-1.86; P < .001). After exclusion of dementia <3 years or > 20 years from baseline/infection, adjusted HR=5.77 (95% CI, 4.92-6.76; P < .001). Risk greater after respiratory, urinary tract, skin, blood and circulatory system, or hospital acquired infections.
Chalitsios et al, 2023, UK[19]	261,976 in Hospital Episode Statistics (HES) data linked to the Clinical Practice Research Database (CPRD).	Retrospective, case control	Mean/SD age, 70.6±17.8 in controls; 69.6±17.8 in exposed.	ICD10 codes for pneumonia in HES data	N	Dementia in CPRD administrative primary care (Read) codes.	Adjusted HR=1.53, 95% CI 1.46-1.61 Stronger associations in younger people: age 45-60 years (p-value for interaction <0.0001)
Chu et al, 2022, Taiwan[20]	11,712 patients with bacterial pneumonia and 11,120 Taiwan National Health Insurance	Retrospective	≥ 45 years Mean age/SD 68.1/11.6 with bacterial pneumonia	Bacterial pneumonia	N	All dementia, Alzheimer's disease (AD), vascular dementia (VaD), and unspecified dementia	All dementia HR=2.83, AD HR=2.44, VaD HR=4.15 unspecified dementia=2.62 adjusted

	Research Database,		and 68.0/11.7 controls			up to 17 years of follow-up	Risks of dementia and VaD were associated with repeated hospitalization due to bacterial pneumonia in a dose-dependent manner.
Douros et al, 2021, UK[21]	4,262,092 Clinical Practice Research Datalink.	Retrospective	Mean age 60.4 years; 52% female	Infection diagnosed >2 years before the index date	N	Dementia from READ codes	AD OR=1.05; 95% CI, 1.02-1.08). Risk increased with time since infection, peaking after 12-30 years (OR, 1.11;1.05-1.17). Risk not increased with cumulative number of infections.
Fritze et al, 2021, Germany[22]	161,567 German health claims data.	Retrospective	65 years or older	Sepsis	N	Dementia from 2006 FU from 2004 to 2015	Increased risk=3.14 (95% CI 2.83-3.49) in non-ICU, Increased risk=2.22 (95% CI: 1.83-2.70) for ICU ***adjusted for delirium***, surgery, age, sex, and comorbidities Risk remitted after 2-years.
Gracner et al, 2021, USA[23]	20,698 Nursing Home residents Medicare data	Retrospective	65 years or older	Infection-related hospitalisation	N	Cognitive function score (1-4, 1=intact, 4=severe impairment) Data from 2011-2017 Analysis 2020-21	CFS decreased by 0.06 (95% CI 0.05-0.07, p<0.001) over the first quarter. Increase greater in those >/=85 years, with Alzheimer's Disease and with sepsis
Hendel et al, 2023, Sweden[24]	712 in the population based SNAC-K study	Prospective, case control	60 years or older	Pneumonia, hospitalised	N	Cognitive decline and dementia by DSM IV criteria	Transient impact on cognition in the first 2.5 years (B = -0.94, 95% CI -1.75, -0.15) but HR for dementia not increased (1.17, 95%CI 0.82, 1.66).
Khairan et al, 2022, Japan[25]	9952 Japan Gerontological evaluation study	Prospective	65 years or older	History of pneumonia >1 year before the baseline questionnaire in 2013	N	Dementia from public long-term care insurance registration FU 2013 to 2019	HR=1.20, 95% CI:0.81-1.78) overall HR=2.30 (1.47-3.62) in those with frailty and pneumonia

Morton et al, 2020, UK [26]	60,392 Stroke patients<br>Clinical Practice Research Datalink	Retrospective	Median (IQR) age 74.3 years<br>51.2% male	Any GP-recorded infection (lower respiratory tract Infection, urinary tract infection, skin and soft tissue infection)	N	Dementia from READ codes occurring from 3 months to 5 years after stroke.	HR=1.44, 95% CI 1.21-1.71)<br>HR 1.84, 95% CI 1.58-2.14 when hospitalised infections were included.<br>strongest in the 3-12 months following stroke<br>Late dementia (1-5 years) only associated with hospitalised, but not with GP-recorded, infections.
Muzambi et al, 2021, UK[27]	989 800<br>Clinical Practice Research Datalink	Retrospective	65 years and older<br>median age 68.6 years<br>54.3% women	Sepsis, pneumonia, other lower respiratory tract infections, urinary tract infections, and skin and soft tissue infections	N	Dementia from READ codes<br>FU Jan 1, 2004, and Dec 31, 2018,<br>Median follow-up 5.2 years (IQR 2.3-9.0)	HR=1.53 (95% CI 1.50-1.55) for any infection (adj for covariates)<br>Sepsis HR 2.08 (1.89-2.29)<br>Pneumonia (HR 1.88, 1.77-1.99)<br>Infections leading to hospital admission HR=1.99 (1.94-2.04).<br>HRs higher in individuals with diabetes vs no diabetes
Muzambi et al, 2022,UK[28]	16,728<br>UKBiobank participants with cognitive data at baseline and follow-up and MR brain imaging	Retrospective	Median age 56 years<br>51% women	History of infection	Y, MRI	Cognitive decline as measured by correct response time, visual memory, fluid intelligence, prospective memory<br>Hippocampal and white matter hyperintensity (WMH) volume	No associations between infections and WMH or hippocampal atrophy<br>No association with cognitive decline
Peters van Ton et al, 2022, Netherlands[29]	331 with infection data<br>Radboud University Nijmegen Diffusion Tensor and Magnetic Resonance Cohort study.	Prospective	50-85 years<br>Mean/SD age 64/8 years, 57% males with cerebral small vessel disease	Infectious events stratified as:<br>severe (e.g., sepsis or hospitalization for infection), mild, or none	Y, MRI	Dementia, cognitive trajectory<br>FU January 2006 and September 2015	HR=1.82; 95% CI, 1.07-3.10; p = 0.027, adjusted age, baseline cognition, and brain volume,<br>Infections had no effect on the trajectory of structural changes to the brain after

							correction for baseline differences.
Sipila et al, 2022, Finland and UK[30]	260 490 primary cohort, Finnish Public Sector study, the Health and Social Support study, the Still Working study and 485 708 replication cohort. UK Biobank	Retrospective	Adults (≥18 years)	Infection in national hospital inpatient registers (using the International Classification of Diseases 10th Revision codes)	N	Dementia from hospital records, medication records, and death certificates. Dec 19, 2006, to Oct 1, 2010, median follow-up 15·4 years	Adjusted HR 1·48 (95% CI 1·37-1·60) and replication cohort (2·60 [2·38-2·83] dose-response association VaD HR=2·09 (95% CI 1·59-2·75) AD HR=1·20 [1·08-1·33], primary cohort VaD HR=3·28 (2·65-4·04) AD HR=1·80 (1·53-2·13) replication cohort)
Wang et al, 2021, USA[31]	21,823 REGARDS	Prospective	Mean/SD age 64.3/9.2 years	Sepsis , defined as emergency department visit and/or hospital admission for infection with at least two Systemic Inflammatory Response Syndrome (SIRS) criteria.	N	Global cognitive function (Six-Item Screener) Incident cognitive impairment, new learning (verbal memory executive function/semantic fluency, animal fluency Median FU 10 years	Sepsis was associated with faster long-term declines in Six-Item Screener (-0.02 points per year faster [95% CI, -0.01 to -0.03]; p < 0.001) and faster long-term rates of incident cognitive impairment (odds ratio 1.08 per year [95% CI, 1.02-1.15]; p = 0.008) compared with presepsis slopes. Adjusted for sociodemographics, health behaviours, chronic medical conditions, depressive symptoms, health status, and select biomarkers
Wu et al, 2022 Taiwan[32]	7,473 patients with pneumonia requiring ICU 22,419 controls matched by sex and age. Taiwan's National Health Insurance Research Database	Retrospective	Mean/SD age 69.2/8.8 years with ICU stay 69.2/9.1 years without ICU stay	Pneumonia	N	Dementia FU 2000 and 2015.	HR=3.37 (95% CI 3.09-3.68) for patients with pneumonia requiring ICU vs control, adjusted

References, Systematic Reviews

1. Goldberg TE, Chen C, Wang Y, et al. Association of Delirium With Long-term Cognitive Decline: A Meta-analysis. JAMA Neurol. 2020;77:1373-1381.
2. Muzambi R, Bhaskaran K, Brayne C, Davidson JA, Smeeth L, Warren-Gash C. Common Bacterial Infections and Risk of Dementia or Cognitive Decline: A Systematic Review. J Alzheimers Dis. 2020;76:1609-1626.
3. Bayer TA, Jiang L, Singh M, et al. Delirium and Subsequent Dementia (ADRD) Diagnosis After Heart Failure Hospitalization. Epidemiology. 2022;70(SUPPL 1):S146-S7.
4. Chu NM, Bae S, Chen X, et al. Delirium, changes in cognitive function, and risk of diagnosed dementia after kidney transplantation. American Journal of Transplantation. 2022;22:2892-902.
5. Giroux M, Emond M, Nadeau A, et al. Functional and cognitive decline in older delirious adults after an emergency department visit. Age & Ageing. 2021;50:135-40.
6. Hayhurst CJ, Marra A, Han JH, et al. Association of Hypoactive and Hyperactive Delirium With Cognitive Function After Critical Illness. Critical Care Medicine. 2020;48:e480-e8.
7. Jaatinen R, Luukkaala T, Hongisto MT, Helminen H, Nuotio MS. In-hospital delirium as a prognostic factor for new cognitive disorder in a 1-year post-hip fracture follow-up. Dementia and Geriatric Cognitive Disorders. 2021;50:296-302.
8. Knaak C, Brockhaus WR, Spies C, et al. Presurgical cognitive impairment is associated with postoperative delirium and postoperative cognitive dysfunction. Minerva Anestesiologica. 2020;86:394-403.
9. Krogseth M, Davis D, Jackson TA, et al. Delirium, neurofilament light chain, and progressive cognitive impairment: analysis of a prospective Norwegian population-based cohort. Lancet Healthy Longev. 2023;4:e399-e408.
10. Kunicki ZJ, Ngo LH, Marcantonio ER, et al. Six-Year Cognitive Trajectory in Older Adults Following Major Surgery and Delirium. JAMA Intern Med. 2023;183:442-450.
11. Leighton SP, Herron JW, Jackson E, Sheridan M, Deligianni F, Cavanagh J. Delirium and the risk of developing dementia: a cohort study of 12 949 patients. Journal of Neurology, Neurosurgery and Psychiatry. 2022;93:822-7.
12. Nerdal V, Gjestad E, Saltvedt I, et al. The relationship of acute delirium with cognitive and psychiatric symptoms after stroke: a longitudinal study. BMC Neurology. 2022;22:234.
13. Racine AM, Touroutoglou A, Abrantes T, et al. Older Patients with Alzheimer's Disease-Related Cortical Atrophy Who Develop Post-Operative Delirium May Be at Increased Risk of Long-Term Cognitive Decline after Surgery. Journal of Alzheimer's Disease. 2020;75:187-99.
14. Richardson SJ, Davis DHJ, Stephan BCM, et al. Recurrent delirium over 12 months predicts dementia: results of the Delirium and Cognitive Impact in Dementia (DECIDE) study. Age Ageing. 2021;50:914-20.
15. Webber C, Milani C, Pugliese M, et al. Long-term cognitive impairment after probable delirium in long-term care residents: A population-based retrospective cohort study. J Am Geriatr Soc. 2023 (online prior to print). doi: 10.1111/jgs.18675.
16. Pendlebury ST, Lovett NG, Thomson RJ, Smith SC. Impact of a system-wide multicomponent intervention on administrative diagnostic coding for delirium and other cognitive frailty syndromes: observational prospective study. Clin Med (Lond) 2020;20:454-464.
17. Ahlstrom B, Larsson IM, Strandberg G, Lipcsey M. A nationwide study of the long-term prevalence of dementia and its risk factors in the Swedish intensive care cohort. Critical Care. 2020;24:548.
18. Bohn B, Lutsey PL, Misialek JR, et al. Incidence of Dementia Following Hospitalization With Infection Among Adults in the Atherosclerosis Risk in Communities (ARIC) Study Cohort. JAMA Netw Open. 2023;6:e2250126.

19. Chalitsios CV, Baskaran V, Harwood RH, Lim WS, McKeever TM. Incidence of cognitive impairment and dementia after hospitalisation for pneumonia: a UK population-based matched cohort study. ERJ Open Res. 2023;9:00328-2022.
20. Chu CS, Liang CS, Tsai SJ, et al. Bacterial pneumonia and subsequent dementia risk: A nationwide cohort study. Brain, Behavior, and Immunity. 2022;103:12-8.
21 Douros A, Santella C, Dell'aniello S, et al. Infectious Disease Burden and the Risk of Alzheimer's Disease: A Population-Based Study. Journal of Alzheimer's Disease. 2021;81:329-38.
22. Fritze T, Doblhammer G, Widmann CN, Heneka MT. Time course of dementia following sepsis in German health claims data. Neurology. 2021;8(1).
23. Gracner T, Agarwal M, Murali KP, et al. Association of Infection-Related Hospitalization With Cognitive Impairment Among Nursing Home Residents. JAMA Network Open. 2021;4:e217528.
24. Hendel MK, Rizzuto D, Grande G, et al. Impact of Pneumonia on Cognitive Aging: A Longitudinal Propensity-Matched Cohort Study. J Gerontol A Biol Sci Med Sci. 2023;78:1453-1460.
25. Khairan P, Shirai K, Shobugawa Y, et al. Pneumonia and subsequent risk of dementia: Evidence from the Japan Gerontological evaluation study. International Journal of Geriatric Psychiatry. 2022;37(11) (no pagination).
26. Morton CE, Forbes HJ, Pearce N, Smeeth L, Warren-Gash C. Association between common infections and incident post-stroke dementia: A cohort study using the clinical practice research datalink. Clinical Epidemiology. 2020;12:907-16.
27. Muzambi R, Bhaskaran K, Smeeth L, Brayne C, Chaturvedi N, Warren-Gash C. Assessment of common infections and incident dementia using UK primary and secondary care data: a historical cohort study. The Lancet Healthy Longevity. 2021;2:e426-e35.
28. Muzambi R, Bhaskaran K, Rentsch CT, et al. Are infections associated with cognitive decline and neuroimaging outcomes? A historical cohort study using data from the UK Biobank study linked to electronic health records. Transl Psychiatry. 2022;12:385.
29. Peters van Ton AM, Meijer-van Leijsen EMC, Bergkamp MI, et al. Risk of Dementia and Structural Brain Changes Following Nonneurological Infections During 9-Year Follow-Up. Critical Care Medicine. 2022;50:554-64.
30. Sipilä PN, Heikkilä N, Lindbohm JV, et al. Hospital-treated infectious diseases and the risk of dementia: a large, multicohort, observational study with a replication cohort. Lancet Infect Dis. 2021;21:1557-67.
31. Wang HE, Kabeto MM, Gray M, et al. Trajectory of Cognitive Decline After Sepsis. Critical Care Medicine. 2021;49:1083-94.
32. Wu CH, Peng CK, Chung CH, Chien WC, Tzeng NS. Real-World Evidence for the Association Between Pneumonia-Related Intensive Care Unit Stay and Dementia. Psychiatry Investigation. 2022;19:247-58.

Brain imaging and rating of white matter disease (WMD) on CT and MRI brain scans

MRI scans were performed on a 1.5-T Philips Achieva scanner, and CT scans on a Toshiba, Aquilion 64, 64-slice scanner. The MRI sequences chosen for evaluation were the transverse T2 and the coronal fluid-attenuated inversion recovery. This latter was only available in coronal view, according to the clinical protocol applied in our hospital, and therefore it was mainly used to support and clarify the T2 findings.

WMD was prospectively and independently coded by a neuroradiologist and in addition, by an experienced neurologist for reproducibility evaluation. Assessments were made blind to clinical data.

WMD was graded according to the following:

1. The ARWMC scale[1] for both CT and MRI, rating 5 different regions in both hemispheres according to a 0–3 score. We used the total score derived from this scale and categorized it into absent (0), mild (1 to 5), moderate (6 to 10), and severe (over 10) WMD (**Figure S1).**
2. A qualitative scale ("Oxford scale") based on the severity score (absent, mild, moderate, or severe) of the Blennow scale[2] for CT scans, and a modified version of the Fazekas scale,[3] considering periventricular and deep white matter lesions altogether, for MRI scans .

The neuroradiologist only applied the Oxford scale, while the neurologist applied both the ARWMC scale and the Oxford scale.

The inter-rater agreement on presence and severity of WMD on CT was assessed by κ statistics in a subset of 996 consecutive cases and for MRI on 100 cases. We also performed an agreement study between CT and MRI in the 416 patients who had had both modalities of imaging, using the SAS software to calculate both simple and weighted kappa. The inter-rater agreement on presence of WMD in 996 consecutive cases imaged by CT was moderate to good (κ = 0.64, 0.59–0.69, for presence of any WMD, and 0.58, 0.55–0.62 for severity). The inter-rater agreement on presence of WMD in 100 consecutive cases imaged by MRI was also good (κ = 0.78, 0.65–0.90 for presence and 0.66, 0.56–0.76 for severity of WMD). In the 416 patients who had both CT and MRI, agreement between independent assessments made on the different modalities was not significantly less than the interobserver reproducibilities of either modality alone.[4]

Figure S1: Moderate/severe WMD on MR brain imaging (top) and on CT brain imaging (bottom)

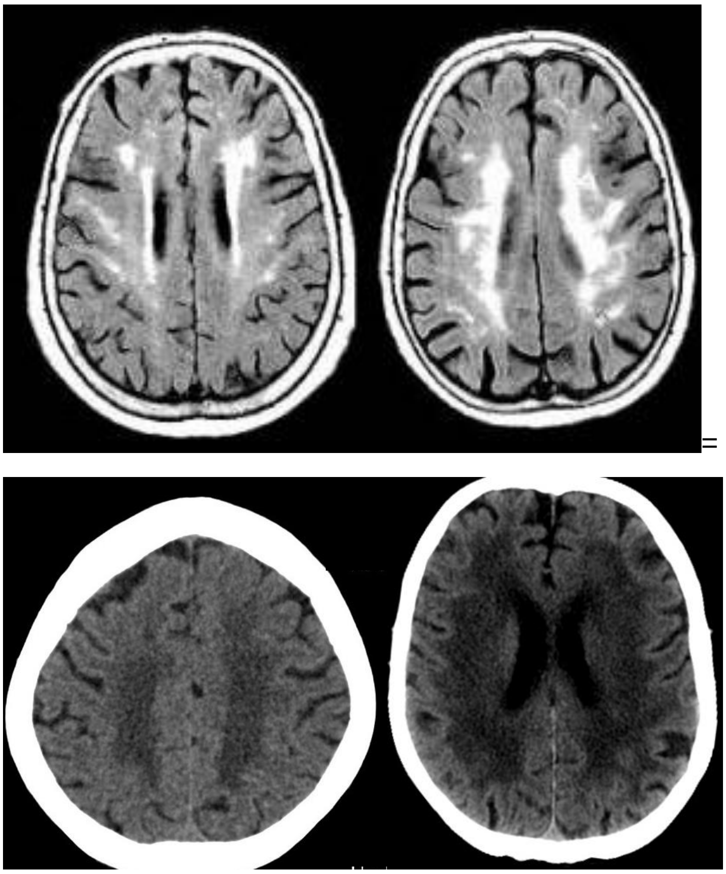

References

1. Wahlund LO, Barkhof F, Fazekas F, Bronge L, Augustin M, Sjögren M et al., on behalf of the European Task-Force on Age-Related White Matter Changes A new rating scale for age-related white matter changes applicable to MRI and CT. Stroke 2001;32:1318-1322.
2. Blennow K, Wallin A, Uhlemann C, Gottfries CG. White matter lesions on CT in Alzheimer patients: relation to clinical symptomatologic and vascular factors. Acta Neurol Scand 1991;83:187-193.
3. Fazekas F, Chawluk JB, Alavi A, Hurtig HI, Zimmerman RA. MR signal abnormalities at 1.5 T in Alzheimer's dementia and normal aging. AJR Am J Roentgenol 1987;149:351-356.
4. Simoni M, Li L, Paul NL, Paul NL, Gruter BE, Schulz UG, Küker W, Rothwell PM. Age- and sex-specific rates of leukoaraiosis in TIA and stroke patients: population-based study. Neurology 2012;79:1215-1222.

Dementia diagnosis

We examined issues around measured dementia diagnosis with reference to the OXVASC methodology in three previous publications in Stroke, specifically the impact of selection[1] and attrition[2] biases and problems interfering with cognitive testing.[3]

In OXVASC, we used multiple methods of follow-up which have been shown to substantially reduce attritional biases in identification of dementia in OXVASC.[2] Dementia was defined as pre- or post-event according to whether the diagnosis was made before or after the index event.[1] Pre-event dementia diagnosis was made using the following information: i) baseline clinical assessment by study physician and discussion with relatives or other informant; ii) any dementia diagnosis, and related consultations and investigations, where available, in the primary care record, with hand-searching of the entire record including individual consultations, clinic letters, and hospitalisation documentation. In many cases, diagnosis was recorded in hospital notes or clinic letters but not in the primary care diagnosis list. In other cases, the diagnosis was made by STP on the basis of cognitive and functional impairment apparent from hand-searching of the medical record including individual primary care consultations or clinical hospital physician, nursing and allied health care professional records.

In patients without pre-event dementia, post-event dementia was diagnosed by STP using the same methodology (i.e. using the baseline and follow-up clinical and cognitive assessment data, supplemented by hand-searching of primary care records to death or 5-year follow-up). MMSE was done at each follow-up interview, and dementia was diagnosed if MMSE was <24 and remained <24 for all subsequent follow-ups in patients in whom cognitive testing was not affected by problems such as poor vision, hemiparesis or depression. A small number of subjects had a MoCA but no MMSE in whom none had dementia. In patients with telephone testing, incomplete testing or inability to perform a cognitive test at study interview (e.g. severe deafness) or with missing study follow-up assessment, dementia was diagnosed by STP on the basis of all available study assessment data and hand-searching of primary care, hospital and death records, based on DSM-IV criteria as described for pre-event dementia.[1,2]

Regarding date of dementia diagnosis, although study interview did not routinely occur between 1 and 5 years after the index event, some patients had data from a study interview during this period because of a recurrent event. In other patients, details of a dementia diagnosis made between 1 and 5-year follow-up were obtained at the 5-year study follow-up and the date of diagnosis was obtained from medical records. For patients who did not have 5-year follow-up (eg because of death or drop-out between 1 and 5 years, untestability, telephone/email follow-up without a cognitive test), all available medical records were reviewed by STP. Where available, the exact date of diagnosis was recorded. If there was no clear date given in records, an approximate date of diagnosis was assigned based on review of study and medical records and information from informants where available.

We did not assess for functional impairment in patients diagnosed with dementia using the MMSE scores partly because it can be difficult attributing functional impairment to cognition versus physical disability in patients with cerebrovascular events. However, we performed sensitivity analyses to check whether this may have affected our results. Thirty-seven patients had low MMSE with a modified Rankin score of <2. In sensitivity analyses, removal of these patients had no significant effect on our findings and specifically, no impact on the relationship between event severity and dementia (HR=1.12 (1.10-1.13) per point increase in NIHSS vs 1.12 (1.10-1.13) adjusted for age, sex and education). Similarly, use of a lower cut-point (MMSE<20) in tested patients, did not change the relationship between event severity and post-event dementia (HR= 1.12 per point increase in NIHSS, p<0.0001).

For cases in which there was uncertainty (mainly in deciding whether cognitive impairment was sufficiently severe pre-event to be classed as pre-event dementia rather than

progressing post-event to dementia), all study and medical records information was reviewed and resolved by discussion between STP and PMR.

For this study, we did not identify Mild Cognitive Impairment (MCI) and such patients were not therefore included in the dementia diagnosis group.

References

1. Pendlebury ST, Chen PJ, Bull L, Silver L, Mehta Z, Rothwell PM; Oxford Vascular Study. Methodological factors in determining rates of dementia in transient ischemic attack and stroke: (I) impact of baseline selection bias. Stroke. 2015;46:641-6.

2. Pendlebury ST, Chen PJ, Welch SJ, Cuthbertson FC, Wharton RM, et al; Oxford Vascular Study. Methodological Factors in Determining Risk of Dementia After Transient Ischemic Attack and Stroke: (II) Effect of Attrition on Follow-Up. Stroke. 2015;46:1494-500.

3. Pendlebury ST, Klaus SP, Thomson RJ, Mehta Z, Wharton RM, Rothwell PM. Methodological Factors in Determining Risk of Dementia After Transient Ischemic Attack and Stroke: (III) Applicability of Cognitive Tests. Stroke. 2015 ;46:3067-73.

Ascertainment of delirium

Delirium diagnosis was made using ICD-10 diagnostic coding available for all admissions supplemented by hand-searching of medical records. Delirium was defined according to ICD-10-based diagnostic coding: A812, E512, F05, F104, F114, F124, F134, F164, F174, F184, F194, G92 and G934.[1] Given that hospital coding of delirium is specific but insensitive, we also identified delirium based on ICD-10 symptom codes: R410, R418, R441, R443 and R451.[1]

During previous work to ascertain dementia in OXVASC TIA and stroke cohorts ascertained 2002-2012, STP had hand-searched OXVASC study records, hospital records and primary care consultations.[2] During the course of these searches, evidence of cognitive impairment of any type was recorded by STP in electronic spreadsheet files including episodes of acute confusion or documented delirium as well as evidence of dementia. Using these records as a starting point, AS undertook a focussed search of relevant hospitalisation and OXVASC study records to determine delirium diagnosis according to the methodology of Kuhn et al. which has been shown to have good accuracy when done by experienced clinicians.[3] In

cases of uncertainty, all the available information was reviewed and discussed by STP and AS together. Delirium diagnosis was made according to the DSM-IV criteria.

Since 2012, STP has led a multicomponent intervention to improve the recognition and documentation of co-existing cognitive frailty in hospital patients for all patients admitted to the Oxford University Hospitals NHS Foundation trust-OUHFT comprising four general hospitals covering the Oxfordshire region with a population of ~660,000).[2] The intervention includes mandatory routine cognitive screening for patients aged ≥70 years or <70 years with confusion/altered behaviour and the OUHFT cognitive screen includes the 10-point abbreviated mental test score (AMTS) together with the Confusion Assessment Method (CAM) for delirium (see Box).[1,4] Screening for incident delirium arising during admission is advised where there is a change in the patient's condition during admission and is therefore performed on an ad hoc basis using the same screening method. Monthly figures on the numbers with a completed cognitive screen are required to be returned to NHS England and are publicly available - *https://www.england.nhs.uk/statistics/statistical-work-areas/dementia/dementia-assessment-and-referral-2019-20*.

The cognitive screen was administered via a structured paper clerking proforma from 2012-2015 and then was thereafter built as an electronic powerform following introduction of electronic patient records in 2015. The multicomponent intervention including cognitive screening has led to a 6-fold improvement in the sensitivity of ICD-10 diagnostic coding for delirium with no loss of the high specificity.[1] Ascertainment of delirium in the later periods of the current OXVASC study follow-up was therefore made easier by these initiatives.

Box: CAM

A. Acute onset/fluctuation – obtained from a detailed collateral history. This is particularly important where a patient has a per-existing cognitive impairment where the change from the previous level of function and behaviour may be key to the recognition of delirium.
B. Inattention- Does the patient have difficulty focusing attention, is the patient easily distractible? Do they have difficulty keeping track of what is being said? Did the patient struggle with 20-1 backwards doing the AMTS or in keeping their mind on the questions?
C. Disordered thinking –. Is the patient's train of thought muddled or illogical? Is it hard to follow what they are saying?
D Altered level of consciousness - Is the patient sleepy, less interactive than usual, or agitated (eg walking around the ward, interfering with equipment, picking at clothes/drips/bedclothes). This is another key aspect of delirium that aids its identification in patients with a pre-existing cognitive impairment. Informants will be often be very skilled at picking up such changes.

Notes: In practice, criterion C is most challenging but criterion D is usually more easily assessed. To screen positive patients must have A and B and either C or D. The diagnosis of delirium may be particularly difficult in patients with pre-existing cognitive impairment. At present, there is no consensus on how best to evaluate these patients but, criteria A and D from the CAM may be particularly useful and a detailed collateral history is key.

In our study, of the 129 admissions identified with delirium, 66 (51%) were identified through routine administrative coding (ICD-10 codes: A812, E512, F05, F104, F114, F124, F134, F164, F174, F184, F194, G92 and G934; and symptom codes: R410, R418, R441, R443 and R451). An additional 63 (49%) admissions with delirium were identified through hand searching of medical records by two clinicians with expertise in delirium.

References

1. Pendlebury ST, Lovett NG, Thomson RJ, Smith SC. Impact of a system-wide multicomponent intervention on administrative diagnostic coding for delirium and other cognitive frailty syndromes: observational prospective study. Clin Med (Lond) 2020;20:454-464.

2. Pendlebury ST, Rothwell PM. Incidence and prevalence of dementia associated with transient ischaemic attack and stroke: analysis of the population-based Oxford Vascular Study. Lancet Neurol 2019;18:248-258.

3. Kuhn E, Du X, McGrath K, Coveney S, O'Regan N, Richardson S, et al. Validation of a consensus method for identifying delirium from hospital records. PLoS One. 2014;9:e111823.

4. Pendlebury ST, Lovett NG, Smith SC, Dutta N, Bendon C, Lloyd-Lavery A et al. Observational, longitudinal study of delirium in consecutive unselected acute medical admissions: age-specific rates and associated factors, mortality and re-admission. BMJ Open 2015;5:e007808.

Table S3. Charlson Comorbidity Index

**Condition**	**Weight score**	**ICD-10 codes**
Acute myocardial infarction	5	I21-I23, I252, I258
Congestive heart failure	13	I50
Peripheral vascular disease	6	I71, I739, I790, R02, Z958, Z959
Cerebrovascular disease	11	G450-G452, G454-G459, G46, I6
Dementia	14	F00-F03, F051
Chronic pulmonary disease	4	J4, J6
Rheumatic disease	4	M05, M060, M063, M069, M32, M332, M34, M353
Peptic ulcer disease	9	K25-K28
Diabetes		
without chronic complications	3	E101, E105, E106, E108, E109, E111, E115, E116, E118, E119, E131, E136, E138, E139, E141, E145, E146, E148, E149
with chronic complications	-1	E102-E104, E107, E112-E114, E117, E132-E134, E137, E142-E144, E147
Hemiplegia or paraplegia	1	G041, G81, G820-G822
Renal disease	10	I12, I13, N01, N03, N052-N056, N072-N074, N18, N19, N25
Any malignancy without metastasis	8	C0-C6, C70-C76, C8, C9
Liver disease	8	K702, K703, K717, K73, K74
Severe liver disease	18	K721, K729, K766, K767
Metastatic solid tumour	14	C77-C79
AIDS	2	B20-B24

For updated weightings, see https://www.nice.org.uk/Media/Default/Standards-and-indicators/National-Library-of-Quality-Indicators/IAP00385%20supporting%20documentation.docx.

Table S4. Hospital admissions on 5-year follow up after minor stroke and TIA in 1,369 patients

	All admissions	Elective admissions	Non-elective admissions
Total patients	1369	1369	1369
Number of patients with ≥1 admission (%)	891 (65%)	295 (22%)	785 (57%)
Infection related	236 (17%)	21 (2%)	223 (16%)
Non-infection related	842 (62%)	286 (21%)	722 (53%)
Delirium related	103 (8%)	7 (1%)	99 (7%)
Total admissions (%)	2,212 (100%)	439 (20%)	1,773 (80%)
Infection related	356 (18%)	22 (5%)	334 (19%)
Non-infection related	1,856 (84%)	419 (95%)	1,437 (81%)
Delirium related	129 (6%)	7 (2%)	121 (7%)
Admissions, incidence rate (95% CI)	0.44 (0.43-0.46)	0.09 (0.08-0.10)	0.36 (0.34-0.37)
Infection related	0.07 (0.06-0.08)	0.004 (0.002-0.006)	0.07 (0.06-0.07)
Non-infection related	0.37 (0.36-0.39)	0.08 (0.08-0.09)	0.29 (0.27-0.30)
Delirium related	0.03 (0.02-0.03)	0.001 (0.000-0.002)	0.02 (0.02-0.03)
Total days in hospital (%)	15,378	2,110	13,268
Infection related	4,730 (31%)	438 (21%)	4,292 (32%)
Non-infection related	10,648 (69%)	1,672 (79%)	8,976 (68%)
Delirium related	1,736 (11%)	130 (6%)	1,606 (12%)
Days in hospital, mean (S.D.)	11.2 (22.9)	1.54 (5.92)	9.69 (20.5)
Infection related	3.46 (13.3)	0.32 (3.59)	3.14 (12.7)
Non-infection related	7.78 (16.5)	1.22 (4.82)	6.56 (15.3)
Delirium related	1.27 (6.63)	0.09 (2.08)	1.17 (6.28)

Table S5. Multivariable associations between hospitalisations with bacterial infection and risk of dementia stratified by baseline WMD.

Hospital admission(s)	No/mild WMC		Moderate/severe WMC	
	HR (95% CI)	p>|z|	HR (95% CI)	p>|z|
Bacterial infection				
No recorded infection or delirium	1.00 (0.79-1.28)	0.97	1.06 (0.84-1.34)	0.62
With infection	0.69 (0.39-1.24)	0.17	1.83 (1.09-3.07)	0.018
With delirium	3.38 (1.88-6.09)	<0.0001	2.51 (1.39-4.56)	0.002
	n=	793	n=	299
Systemic bacterial infection				
No recorded infection or delirium	0.95 (0.76-1.19)	0.50	1.10 (0.89-1.36)	0.37
With infection	1.00 (0.43-2.35)	0.92	2.23 (1.02-4.89)	0.038
With delirium	3.24 (1.79-5.86)	0.0001	3.15 (1.81-5.48)	<0.0001
	n=	793	n=	299
Localised bacterial infection				
No recorded infection or delirium	1.00 (0.79-1.26)	1.00	1.07 (0.85-1.34)	0.56
With infection	0.56 (0.26-1.20)	0.14	1.63 (0.89-2.97)	0.11
With delirium	3.38 (1.90-6.02)	<0.0001	2.72 (1.50-4.94)	0.0011
	n=	793	n=	299

Figure S2. Associations, after controlling for time-dependent confounding using marginal structural models, between hospitalisation with infection or delirium and risk of dementia stratified by baseline WMD on brain imaging

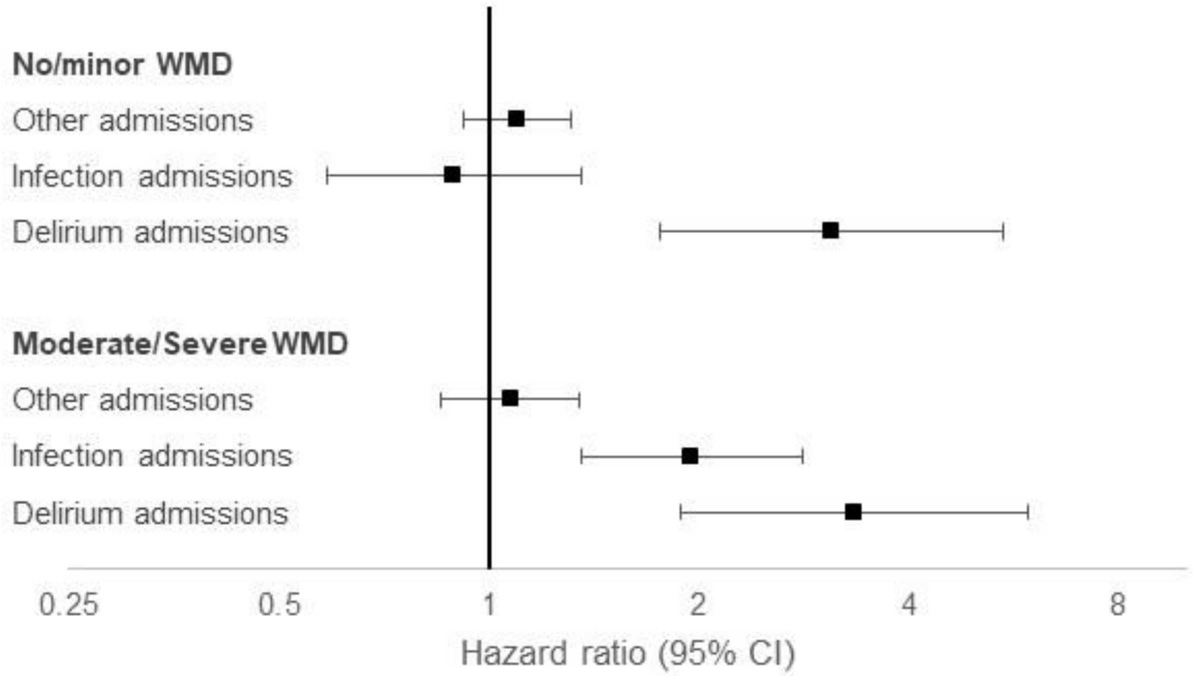

Figure S3. Associations, after multivariable adjustment, between hospitalisations with infection (by type) or delirium and risk of dementia stratified by baseline WMD on brain imaging.

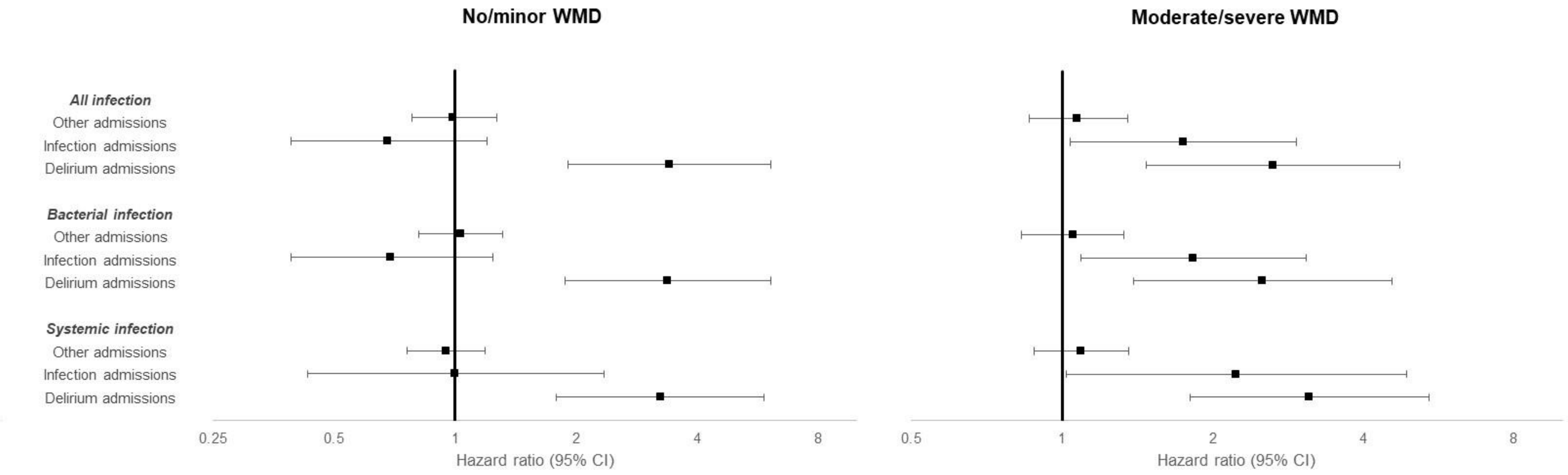

Supplement: Supplementary Appendix [file EMS207692-supplement-Supplementary_Appendix.pdf]
